# Residues 27T and 297A in VP2 contribute to the enhanced replication and pathogenicity of raccoon dog parvovirus

**DOI:** 10.1128/jvi.01012-25

**Published:** 2025-09-05

**Authors:** Liwen Xu, Wenyu Cao, Yawen Liu, Bo Hu, Chengqi Zhang, Shuangshuang Li, Jiajia Liu, Jianhai Fu, Zichuang Zhang, Yajie Sun, Guanyu Zhao, Xiaoyu Deng, Rongguang Lu, Xue Bai

**Affiliations:** 1 Key Laboratory of Special Animal Epidemic Disease, Ministry of Agriculture, Chinese Academy of Agricultural Sciences, Institute of Special Animal and Plant Sciences 243819 , Changchun, China; 2 China Animal Health and Epidemiology Center 499141, Qingdao, China; 3 Institute of Infectious Diseases, Shenzhen Bay Laboratory 551667, Shenzhen, China; Cornell University Baker Institute for Animal Health, Ithaca, New York, USA

**Keywords:** parvovirus, capsid, mutation, pathogenicity, replication, receptor

## Abstract

Raccoon dog parvovirus (RDPV) is a highly contagious pathogen causing severe hemorrhagic enteritis that is fatal in young raccoon dogs. Since 2016, epidemiological investigations have documented recurrent outbreaks of RDPV, exhibiting heightened virulence; however, the molecular mechanisms driving this increased pathogenicity remain poorly understood. In this study, an alignment of 67 complete RDPV sequences identified two high-frequency amino acid mutations at positions 27 and 297 in the VP2 capsid protein that distinguish RDPV strains from before and after the 2016 outbreak. We generated a series of variant viruses with single or double amino acid substitutions using infectious cDNA clones from RDPV/CL/2016 (rRDPV-16) and RDPV/FLD/2010 (rRDPV-10) strains. In F81 cells, the mutant virus derived from rRDPV-16 exhibited reduced replication capacity and attachment than the parental strain, whereas mutants derived from rRDPV-10 showed enhanced replication and attachment. Cell-binding assay, ELISA, and biolayer interferometry (BLI) assays demonstrated a significant decrease in binding capacity to the transferrin receptor (TfR) for the rRDPV-16-derived mutant viruses. In raccoon dogs, these mutant viruses displayed reduced pathogenicity, as evidenced by clinical symptoms, viral loads in rectal swabs, and histopathological changes, compared with the parental strain. Molecular docking and dynamics simulations further suggest that the mutation at position 297 may alter the structural stability of VP2 and affect its binding affinity to raccoon dog TfR (RDTfR). This study identifies amino acid residues at positions 27 and 297 in VP2 as crucial determinants of RDPV replication and pathogenicity, providing insights for the development of therapeutic and prognostic strategies.

**IMPORTANCE:**

Raccoon dog parvovirus (RDPV), a variant of canine parvovirus 2 (CPV-2), has become an emerging threat to fur-bearing canid populations, causing severe disease outbreaks and economic losses. Despite its significance, the molecular basis underlying the enhanced pathogenicity of recent RDPV strains remains poorly understood. Our study identifies two key amino acid mutations (S27T and S297A) in the capsid protein VP2 that significantly increase viral replication, receptor binding affinity, and disease severity in raccoon dogs. These findings elucidate critical determinants of host adaptation and virulence in RDPV, highlighting the evolutionary dynamics of parvoviruses and offering a foundation for targeted antiviral strategies and disease control in fur animal farming.

## INTRODUCTION

Raccoon dog parvovirus (RDPV), a member of the Canine Parvovirus 2 (CPV-2) group, is a non-enveloped, single-stranded DNA virus with a symmetrical capsid composed of 60 units of the capsid protein VP2 (1). VP2, a primary structural protein accounting for approximately 90% of the total protein content, adopts a three-dimensional structure characterized by spikes at the 3-fold axes, canyons at the 5-fold axes, and depressions at the 2-fold axes (2–4). This protein plays a pivotal role in the virus’s entry, stability, and interaction with host cells. CPV infects its hosts by binding to transferrin receptor type 1 (TfR), with the viral capsid interacting specifically through a region near VP2 residue 300 (5, 6). Mutations at VP2 positions 300 and 389 have been shown to significantly affect viral replication and pathogenicity (7). Notably, a single mutation at position 300 of VP2 can dramatically alter receptor binding and infectivity, highlighting the crucial role of VP2 mutations in shaping the virus’s characteristics (8).

RDPV is one of the pathogens responsible for diseases in canid species (9–11). It presents significant challenges to eradication efforts and leads to considerable economic losses in the fur industry (12–14). The earliest documented outbreak of RDPV in China dates back to 1997, confirming its classification within the CPV-2 subtype, with specific amino acid mutations such as S27T, S297A, I219V, and I418T (15). Over the next decade, RDPV was sporadically detected in major fur-farming regions of China. However, since 2016, there has been a marked increase in transmission, accompanied by high fever, severe diarrhea, and mortality (16). As the virus continues to evolve, the CPV-2a variant in raccoon dogs has emerged in recent years in China (15, 17, 18). CPV is also continuously expanding its host range (19). In our study, we compared the virulence of the RDPV/CL/2016 strain, isolated after 2016, with that of the RDPV/FLD/2010 strain, isolated in 2010. Our findings show that the RDPV/CL/2016 strain induces more severe diarrhea, higher mortality, more pronounced organ pathology, and an increased viral load in raccoon dogs (unpublished data).

To further investigate the underlying factors contributing to the enhanced pathogenicity of RDPV, we analyzed the sequences of the major structural protein VP2 (*n* = 67) from all RDPV strains collected in China between 2009 and 2020. Two consistent amino acid mutations at positions 27 and 297 were identified. Position 27, located at the N-terminus of VP2, is typically either hidden or completely unexposed on the surface of native viral particles. However, it may become exposed following specific conformational changes in the viral particle, such as after binding to host cell receptors (20). The S27T mutation was found in the majority of sequences and is currently observed exclusively in RDPV (16, 21), although studies on this mutation remain limited. Position 297 lies within the loop region of VP2, which interacts with the receptor, and is spatially close to the surface-exposed spike at position 300 on the capsid protein (5, 8). This region is under significant selective pressure and may be linked to local adaptation (22–24). The potential contribution of mutations at positions 27 and 297 to increased virulence and pathogenicity remains to be determined.

To investigate the relationship between mutations at positions 27T and 297A and the enhanced pathogenicity of RDPV, we employed reverse genetics to generate a series of mutant viruses by substituting single or double amino acids between the RDPV/CL/2016 and RDPV/FLD/2010 strains. Our results demonstrated that the residues 27T and 297A significantly increased the replication capacity, pathogenicity, and the binding affinity of VP2 to the RDTfR. These findings provide valuable insights into the molecular mechanisms driving RDPV replication and pathogenicity, laying the foundation for the development of more effective therapeutic and prognostic strategies against the virus.

## RESULTS

### Discovery of conserved amino acid mutations in VP2

**Fig 1:**
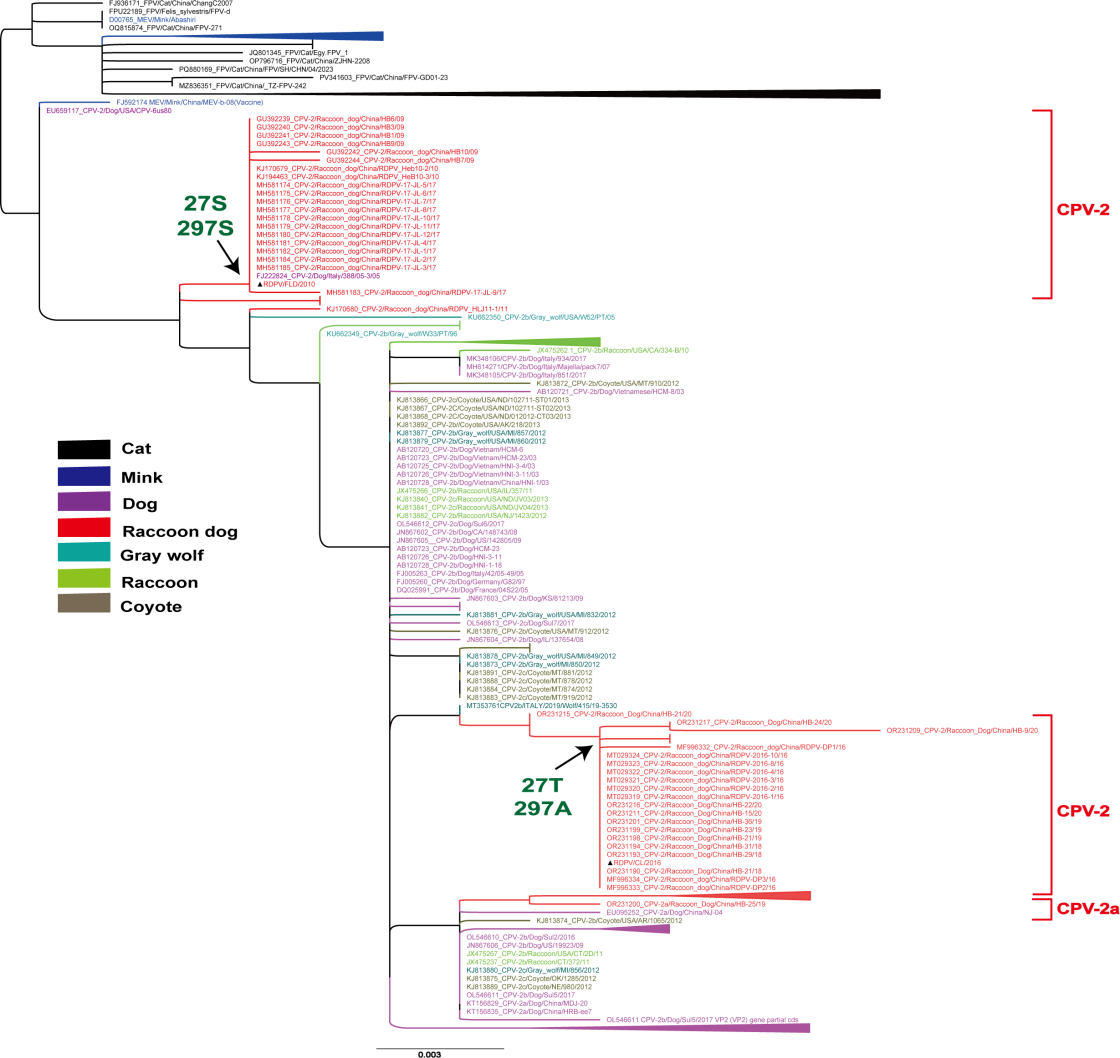
Phylogenetic analysis of VP2 gene sequences from cats, minks, raccoon dogs, gray wolves, raccoons, coyotes, and dogs. A maximum-likelihood (ML) tree was constructed using VP2 gene sequences from 212 parvoviruses. Viruses from different hosts are represented by distinct colors. The antigenic properties of CPV-2 and CPV-2a from raccoon dogs were categorized accordingly. CPV-2 sequences from raccoon dogs, as obtained in this study, are marked with the symbol ▲. GenBank accession numbers: RDPV/CL/2016 (PQ807006); RDPV/FLD/2010 (PQ807007).

Several wild carnivores are key host species for the persistence of CPV in the wild, maintaining the virus transmission chain independent of domestic dogs (25). Early studies reported the detection of CPV in Finnish raccoon dogs, providing historical evidence for the presence of CPV in this host species and further supporting their long-standing susceptibility to CPV infection (26, 27). Phylogenetic analysis of 67 RDPV VP2 sequences revealed that the 27 T-297A and 27 S-297S variants were situated on two distinct branches, with RDPV/CL/2016 and RDPV/FLD/2010 serving as representative strains, respectively (Fig. 1). The phylogenetic tree also indicates that CPV has undergone multiple cross-species transmission events, followed by host-specific adaptive evolution within raccoon dog populations in fur-farming environments, ultimately leading to the emergence of a distinct RDPV lineage. To date, the CPV-2a subtype has been identified in raccoon dogs (1). Multiple sequence alignment of full-length VP2 amino acid sequences revealed distinct mutation hotspots at positions 27 and 297 (Fig. S1). Within the CPV-2 subgroup, all RDPV/CL/2016-like strains (30/30; 100%) consistently harbor threonine (T) at position 27 and alanine (A) at position 297, whereas RDPV/FLD/2010-like strains predominantly feature serine (S) at both positions (Table 1). The 27 T-297A branch is hypothesized to represent an intermediate stage in the evolutionary development of CPV-2 in raccoon dogs.

**TABLE 1:** Amino acid distribution at positions 27 and 297 of CPV VP2 in raccoon dogs across different subgroups

Subgroup	Feature	Representative strain	No. of strains	No. (%) with the amino acid at position:			
				27		297	
				T	S	A	S
CPV-2	27T 297A	RDPV/CL/2016	30	30 (100)	0	30 (100)	0
CPV-2	27S 297S	RDPV/FLD/2010	21	0	21 (100)	3 (14.3)	18 (85.7)

### Construction of infectious cDNA clones

**Fig 2:**
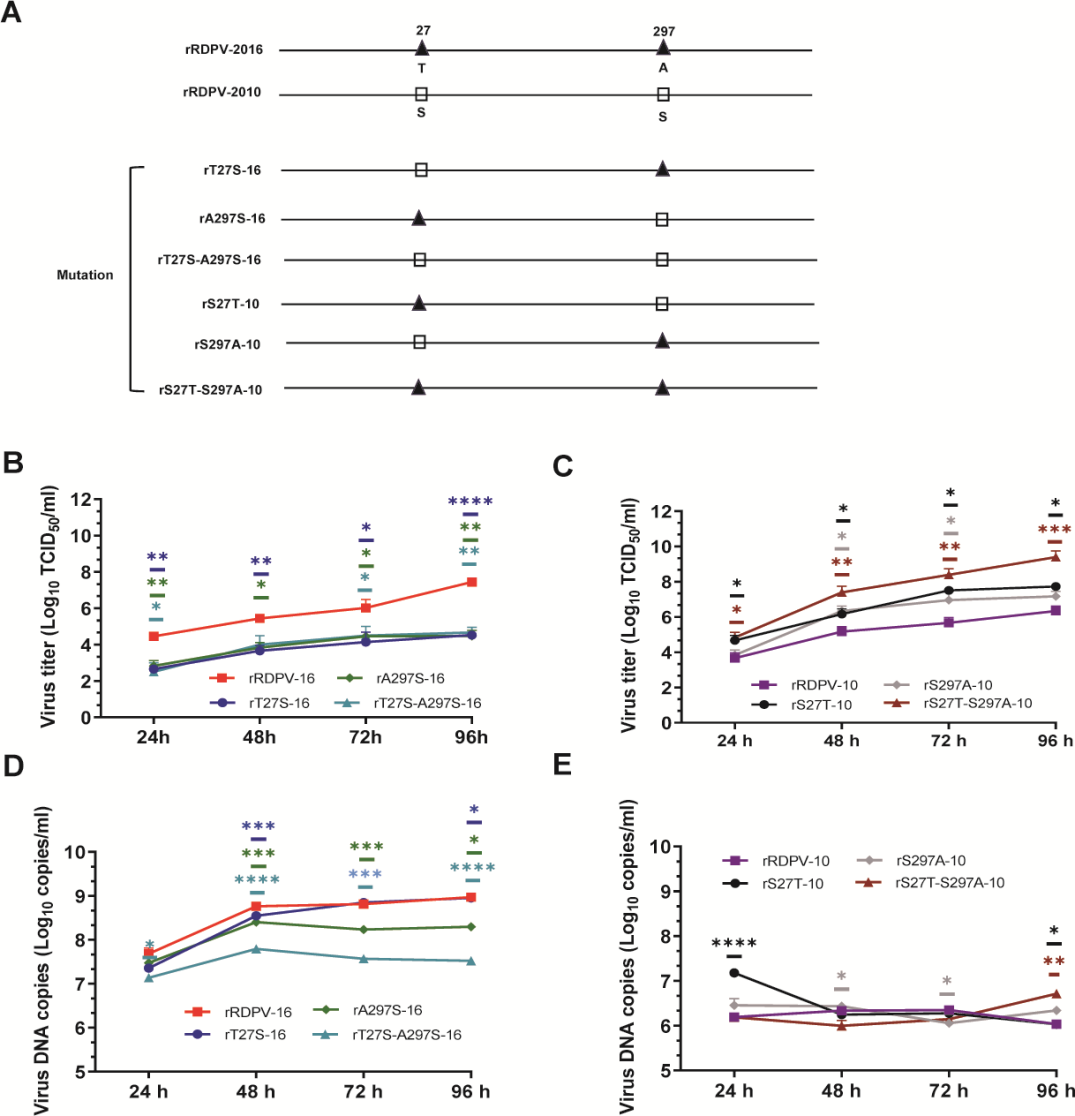
Construction and replication analysis of amino acid mutant viruses. (**A**) The amino acid residues at positions 27 and 297 were swapped between the rRDPV-2016 and rRDPV-2010 sequences. Mutant amino acids in rRDPV-2016 and rRDPV-2010 were represented by triangles and squares, respectively. (**B and C**) Viral growth kinetics. Reverse cDNA versions of rRDPV-16 and rRDPV-10 viruses, containing mutations rT27S-16, rA297S-16, rT27S-A297S-16, rS27T-10, rS297A-10, and rS27T-S297A-10, were used to infect F81 cells at an MOI of 0.01. Viral samples were collected at 24, 48, 72, and 96 h post-infection (hpi) and quantified using a TCID_50_ assay. (**D and E**) Virus DNA copy number. The same viruses were used to infect F81 cells at an MOI of 0.01. Samples were collected at 24, 48, 72, and 96 hpi and quantified by qPCR.

Two infectious cDNA clones, pRDPV-16 and pRDPV-10, were constructed and validated based on the sequences of the wild-type strains (RDPV/CL/16 and RDPV/FLD/2010). Transfection of cells with pRDPV-16 and pRDPV-10 led to detectable viral production, as confirmed by cytopathic effects and immunofluorescence staining (Fig. S2). EcoRI digestion of the cDNA clones pRDPV-16 and pRDPV-10 revealed two distinct bands (3,310 bp and 4,686 bp) in the rescued strains, corresponding to the introduction of a novel EcoRI restriction site, whereas only a single band (7,796 bp) was observed in the parent strains. A total of six mutant viruses were engineered: three from rRDPV-16 (rT27S-16, rA297S-16, and rT27S-A297S-16) and three from rRDPV-10 (rS27T-10, rS297A-10, and rS27T-S297A-10), by introducing amino acid substitutions at residues 27 and 297 of VP2. These mutant viruses were successfully rescued (Fig. 2A).

### Replication advantage of rRDPV-16 linked to mutations at positions 27T and 297A in VP2

In F81 cells, mutant viruses derived from the rRDPV-16 backbone exhibited significantly lower viral titers compared with rRDPV-16 over a time course from 24 to 96 h post-infection (hpi) at a multiplicity of infection (MOI) of 0.01 (Fig. 2B). These mutant viruses consistently showed a reduction of approximately 2.0 log units in viral titers at 24, 72, and 96 hpi relative to rRDPV-16-infected cells. In contrast, mutant viruses derived from the rRDPV-10 backbone (rS27T-10, rS297A-10, and rS27T-S297A-10) exhibited enhanced replication compared with their parental strain. Among these, rS27T-S297A-10 demonstrated significantly higher viral titers than rRDPV-10 at both 72 and 96 hpi (Fig. 2C). Similar results were observed in CRFK cells (Fig. S3); it is noteworthy, however, that viral yields differed significantly between the two cell lines, suggesting that intrinsic cellular differences may influence viral replication efficiency. DNA copy number analysis in F81 cells at 24, 48, 72, and 96 hpi revealed that mutant viruses from the rRDPV-16-derived backbone had lower DNA copy numbers compared with rRDPV-16, with statistically significant differences observed at 48 and 96 hpi (Fig. 2D). Conversely, mutant viruses derived from rRDPV-10 showed the opposite trend in F81 cells, with rS27T-S297A-10 producing significantly higher DNA copy numbers than rRDPV-10 at 96 hpi (Fig. 2E). These findings indicate that rRDPV-16 has a replication advantage, which is closely associated with the presence of 27T and 297A residues in the VP2 protein.

### Residues 27T and 297A enhance the binding capacity of virus to RDTfR

**Fig 3:**
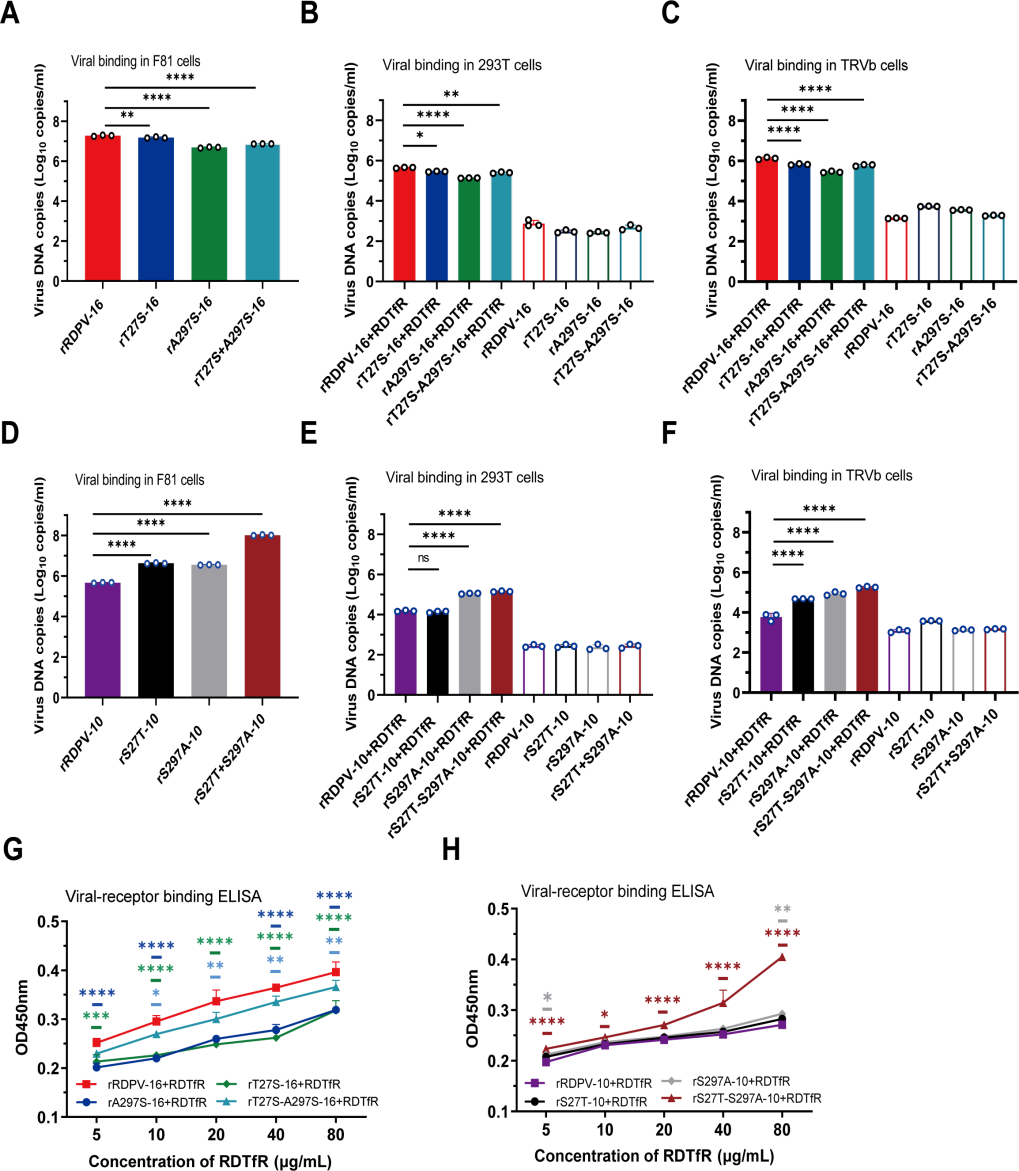
Detecting the binding capacity of the virus with the RDTfR. (**A and D**) RDPV (40 µg/mL) was incubated with chilled F81 cells at 4°C for 2 h. Unbound virus was washed off, and RDPV DNA levels were quantified using qPCR. The binding capacity of RDPV to RDTfR expressed on the cell membrane of transfected HEK 293T (**B and E**) and TRVb (**C and F**) cells was assessed by qPCR. The open bar graphs represent the binding to non-transfected HEK 293T and TRVb cells. (**G and H**) The interaction between RDPV and recombinant RDTfR protein was further evaluated using an ELISA-based binding assay.

We assessed the effect of residues 27T and 297A on the binding of VP2 to RDTfR using several methods, including cell-binding assays, virus receptor ELISA, and BLI. These assays confirmed that the mutations at positions 27T and 297A significantly increased the binding ability of VP2 to RDTfR. Equal mass concentrations of purified viruses (Fig. S4 and S5), including rRDPV-16, rRDPV-10, and the respective mutant viruses, were used to compare their cell-binding capacities. qPCR analysis revealed that rRDPV-16 exhibited approximately 1.2-fold, 3.89-fold, and 2.75-fold higher binding capacities to F81 cells compared with rT27S-16, rA297S-16, and rT27S-A297S-16, respectively (Fig. 3A). Additionally, the binding capacity of rS27T-S297A-10 to F81 cells was significantly greater than that of rRDPV-10, rS27T-10, and rS297A-10 (Fig. 3D). Immunofluorescence assays confirmed the surface expression of transfected RDTfR in HEK 293T (Fig. S6A) and TRVb cells (Fig. S6B), and demonstrated that the virus specifically binds to RDTfR on the cell membrane. Consistently, qPCR analysis showed that the amount of virus bound to RDTfR correlated well with its binding capacity observed in F81 cells (Fig. 3B, C, E and F). Receptor-binding ELISA showed a reduction in the binding affinity of rT27S-16, rA297S-16, and rT27S-A297S-16 to RDTfR compared with rRDPV-16 (Fig. 3G). In contrast, the rS27T-S297A-10 virus exhibited significantly higher binding affinity to RDTfR (Fig. 3H).

**Fig 4:**
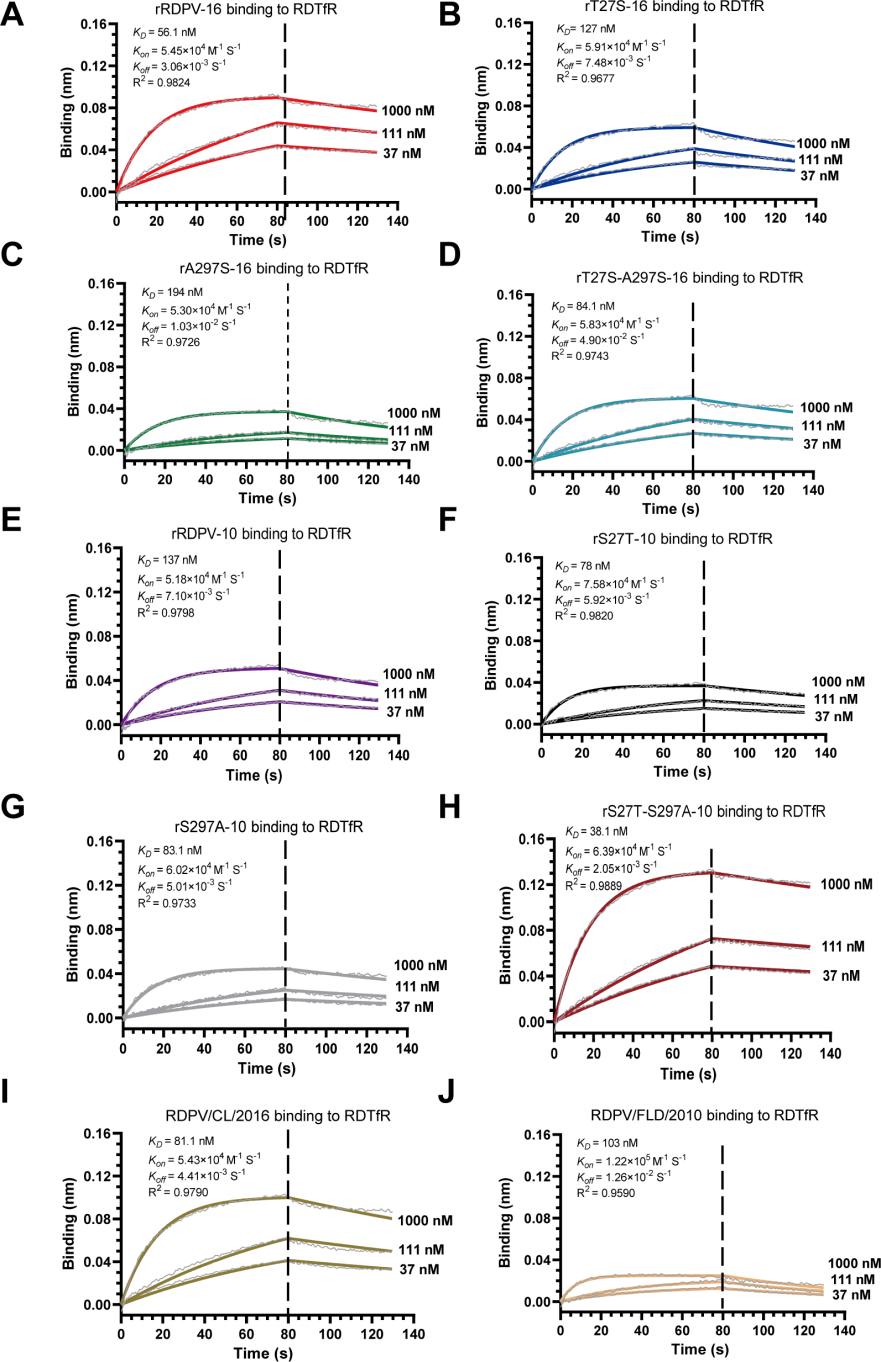
Binding kinetics of rescued viruses to RDTfR. (**A through J**) The binding kinetics of RDPV to RDTfR were measured using the Gator Prime platform (Gator Prime, Silicon Valley, USA). RDPV was captured on the APS probe, followed by the application of serial dilutions of RDTfR protein over the probe surface. The experiments were performed in triplicate, yielding consistent results, with one set of data presented as a representative example.

We employed BLI to assess how residues 27T and 297A impact virus binding to RDTfR. Viruses with various mutations were immobilized on APS biosensors and exposed to three concentrations of purified RDTfR to evaluate their binding abilities. Global fit plots showed binding affinities of 56.1 nM (association constant [*k_on_*] of 5.45 × 10^4^ 1/M·s; dissociation constant [*k_off_*] of 3.06 × 10^−3^ 1/s) for rRDPV-16 (Fig. 4A). Additional rT27S-A297S-16 efficiently bound to RDTfR with a binding affinity of 84.1 nM (*k_on_* of 5.83 × 10^4^ 1/M·s; *k_off_* of 4.90 × 10^−2^ 1/s) (Fig. 4D). However, as shown in the results of Fig. 3A through C, rT27S-16 and rA297S-16 exhibited low binding affinity (Fig. 4B and C). The binding affinities were 137 nM (*k_on_* of 5.18 × 10^4^ 1/M·s; *k_off_* of 7.10 × 10^−3^ 1/s) for rRDPV-10 to RDTfR (Fig. 4E). Notably, rS27T-S297A-10 displayed a higher binding affinity for RDTfR, comparable with that of rRDPV-16 (Fig. 4H), surpassing rS27T-10 and rS297A-10 (Fig. 4F and G). The binding affinities of wild-type RDPV/CL/2016 and RDPV/FLD/2010 to RDTfR were 81.1 nM and 103 nM, respectively (Fig. 4I and J). These findings suggest that mutations at positions 27 and 297, particularly the substitution of serine to threonine at position 27 (S27T) and serine to alanine at position 297 (S297A), substantially enhance the binding affinity of RDPV for RDTfR.

### Double mutations at 27T and 297A enhance viral pathogenicity in raccoon dogs

**Fig 5:**
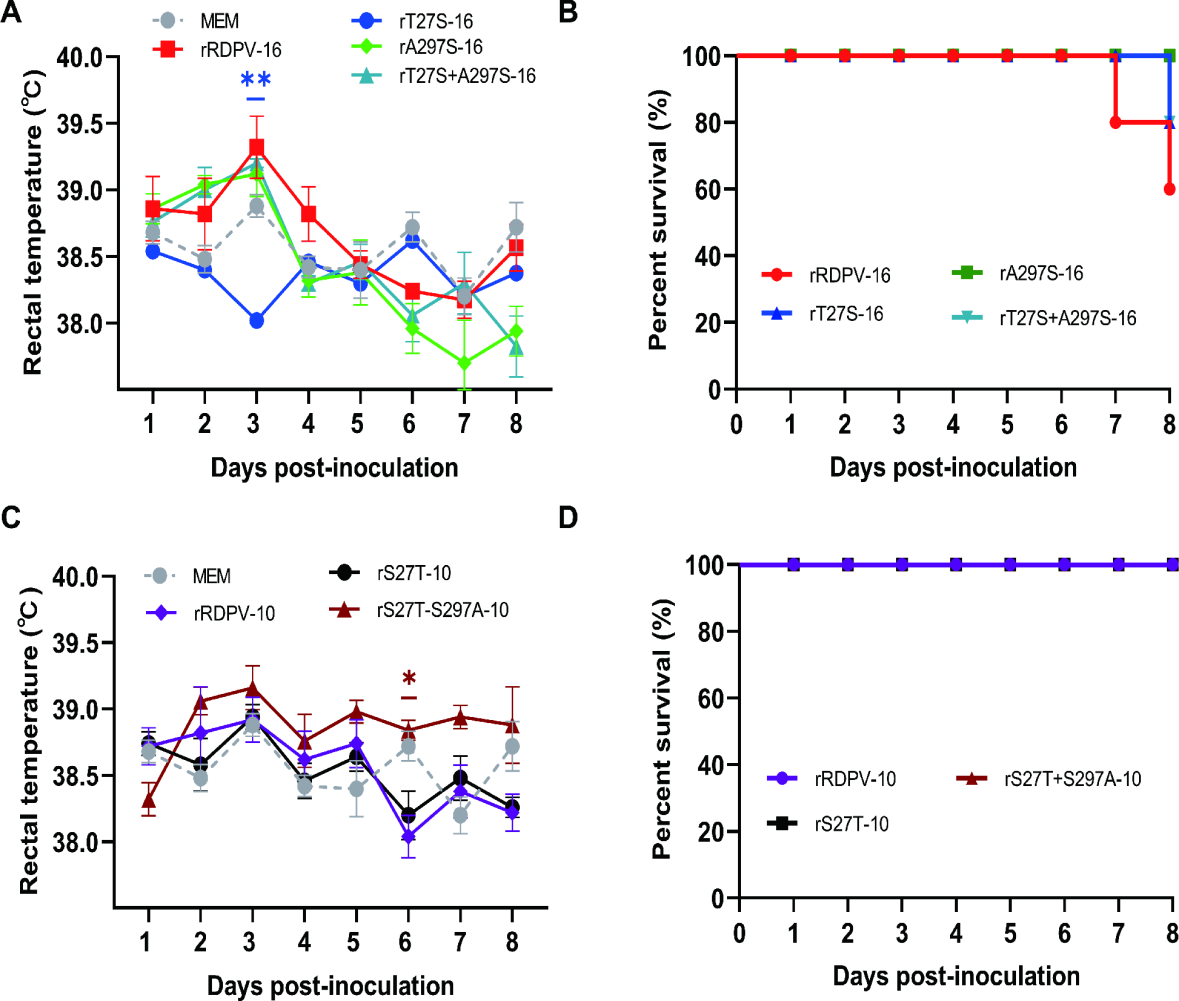
Body temperature and survival tests. (**A and C**) Body temperatures of raccoon dogs inoculated with RDPV. (**B and D**) Survival rates of raccoon dogs following RDPV inoculation. Error bars represent the standard error of the mean (SEM).

**Fig 6:**
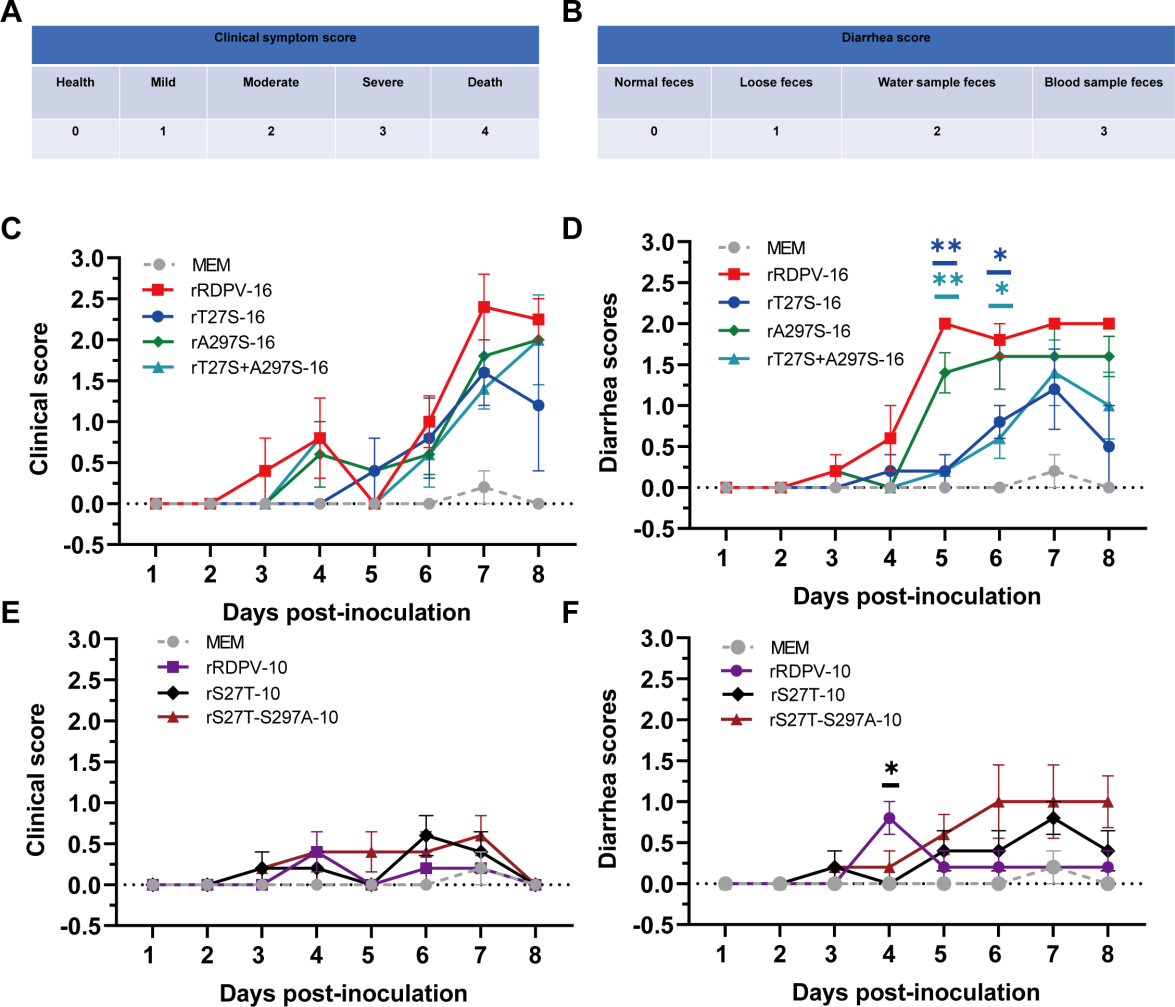
Clinical symptom and diarrhea scores in raccoon dogs inoculated with RDPV. (A, C, and E) Clinical symptom scores of raccoon dogs inoculated with RDPV. Clinical symptoms were scored based on the severity of lethargy, appetite, ocular discharge, and vomiting. (B, D, and F) Diarrhea scores of raccoon dogs following RDPV inoculation. Error bars represent the standard error of the mean (SEM).

To evaluate the virulence and pathogenicity of rRDPV-16 *in vivo*, 2-month-old raccoon dogs were inoculated with 10^5.0^ TCID_50_/mL of rRDPV-16, rT27S-16, rA297S-16, rT27S-A297S-16, rRDPV-10, rS27T-10, or rS27T-S297A-10. Body temperature, mortality, clinical symptoms, and diarrhea were monitored daily for 8 days. Raccoon dogs infected with rRDPV-16 showed a rapid increase in body temperature, accompanied by a 40% mortality rate (2/5) (Fig. 5A and B). In contrast, infections with rT27S-16 and rT27S-A297S-16 resulted in a 20% mortality rate (1/5), whereas no mortality was observed in animals infected with rA297S-16. Furthermore, raccoon dogs infected with rS27T-S297A-10 exhibited elevated body temperatures from 2 to 8 days post-infection (dpi) compared with rRDPV-10, although no mortality was recorded (Fig. 5C and D). Clinical and diarrhea symptom scores are shown in Fig. 6A and B, respectively. The stool morphology scores are shown in Fig. S7A. Although clinical symptoms were more severe in raccoon dogs infected with rRDPV-16, no significant differences were observed when compared with the other mutant virus groups (Fig. 6C). Diarrhea symptoms were significantly more pronounced in the rRDPV-16 group at 5–6 dpi compared with the rT27S-16 and rT27S-A297S-16 groups (Fig. 6D). However, no significant variations in clinical or diarrheal symptoms were observed between rRDPV-10 and the mutant virus groups post-infection in raccoon dogs (Fig. 6E and F). Notably, at 5–8 dpi, diarrheal symptoms were more severe in raccoon dogs infected with rS27T-S297A-10 compared with those infected with rRDPV-10. These results suggest that the double mutations at positions 27T and 297A contribute to the increased virulence and pathogenicity of rRDPV-16 in raccoon dogs.

### Double mutations at 27T and 297A enhance viral replication in raccoon dogs

**Fig 7:**
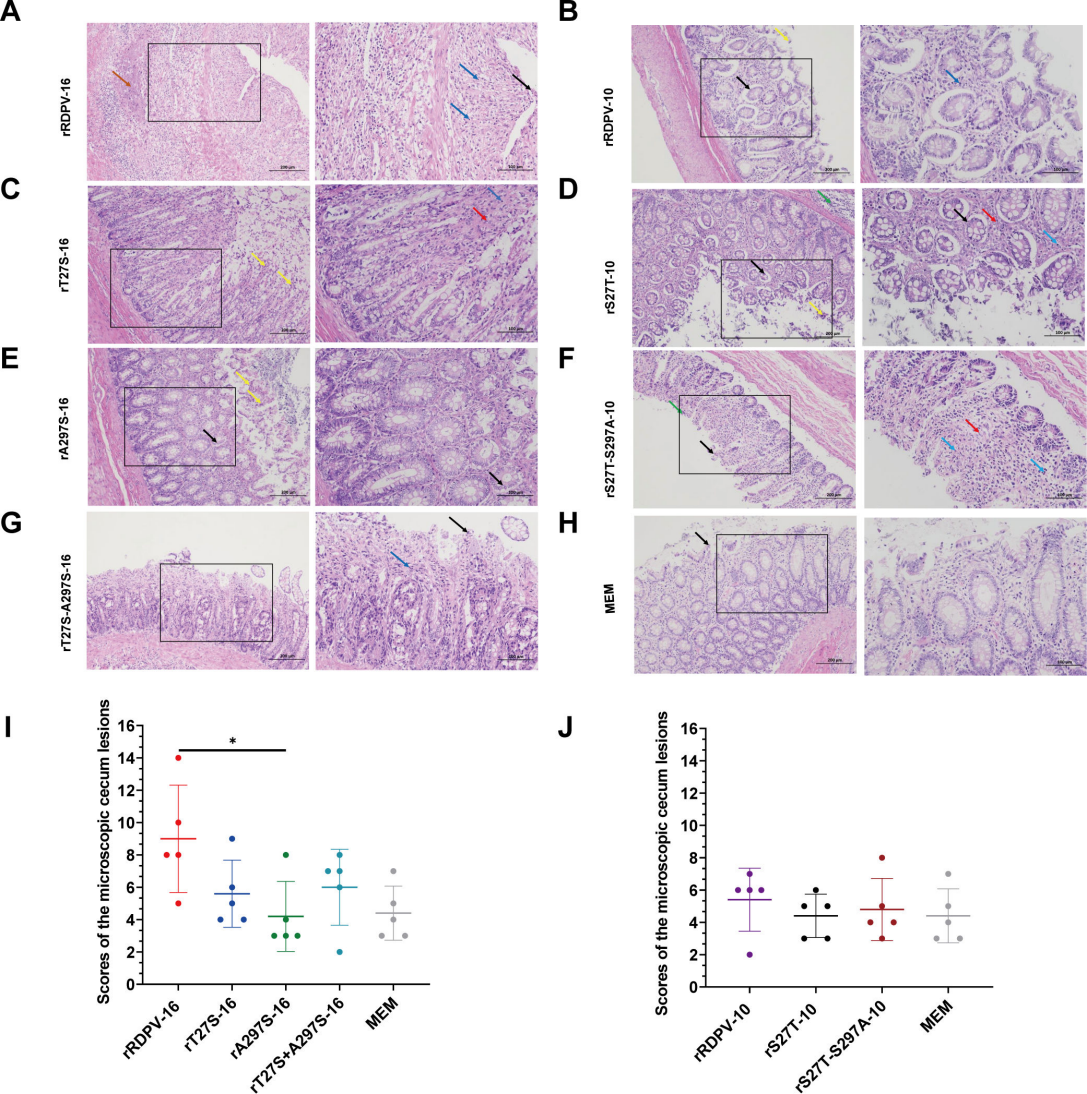
Microscopic cecum lesion scores. (**A through H**) Microscopic examination of the cecum from virus-infected and uninfected control samples. Brown arrow: necrotic foci appear in the submucosal layer. Black arrow: epithelial cells of the mucosal layer and intestinal gland cells are shed. Blue arrow: lymphocyte infiltration. Yellow arrow: necrosis of epithelial cells in the mucosal layer. Red arrow: proliferation of connective tissue in the lamina propria. Green arrow: lymph nodes are visible in the submucosal layer. Images were stained with H&E and magnified at ×100 (left) and ×200 (right). The enlarged area is highlighted within the larger box. (**I and J**) Average scores for the severity of microscopic cecum lesions. Lesion severity, including cell shedding, necrosis, erosion or ulceration, and inflammatory cell infiltration, was graded on a scale from 0 to 4: 0 = normal; 1 = very mild; 2 = mild; 3 = moderate; and 4 = severe.

Macroscopic examination of lesions revealed that raccoon dogs infected with rRDPV-16 experienced more severe intestinal damage compared with those infected with mutant viruses (Fig. S7B). Lesions of varying severity were observed in the intestines of raccoon dogs infected with rRDPV-10 and the mutant viruses (Fig. S1C). Histopathological analysis demonstrated that rRDPV-16-infected raccoon dogs exhibited significant intestinal changes, including mucosal epithelial cell necrosis, shedding, ulceration, connective tissue proliferation, and inflammatory cell infiltration in the lamina propria (Fig. 7A). In contrast, raccoon dogs infected with other viruses showed milder histopathological alterations, including less severe lesions such as mucosal epithelial cell necrosis, shedding, and inflammatory cell infiltration in the lamina propria (Fig. 7B through G). The average histopathological scores for the rRDPV-16 and rA297S-16 groups differed significantly (Fig. 7I). No significant differences were observed in the other infection groups (Fig. 7J).

**Fig 8:**
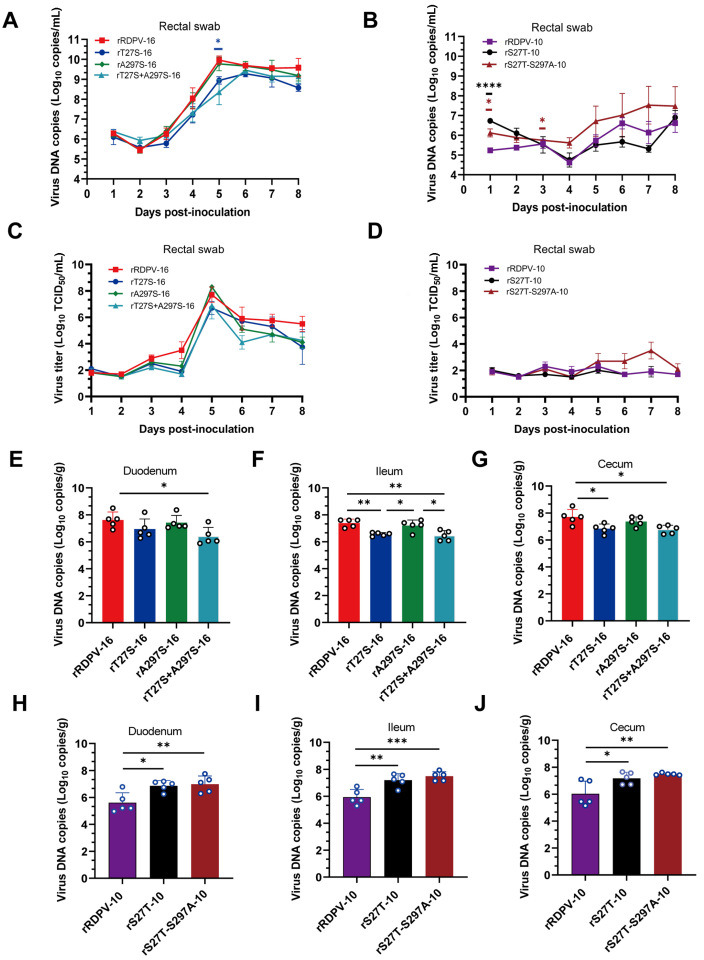
Measurement of virus load in rectal swabs and gastrointestinal tissues (duodenum, ileum, and cecum). Rectal swab DNA copy numbers (**A and B**) and virus titers (**C and D**) in raccoon dogs inoculated with RDPV. Significant differences were observed compared with rRDPV-16 or rRDPV-10. (**E through J**) DNA copy numbers in the duodenum, ileum, and cecum of raccoon dogs inoculated with RDPV.

Viral DNA copy number analysis from rectal swabs showed a slight increase in viral DNA levels in the rRDPV-16 group compared with the rT27S-16, rA297S-16, and rT27S-A297S-16 groups between 4 and 8 dpi, although the difference was not statistically significant (Fig. 8A). In contrast, the rRDPV-10 group exhibited lower viral DNA copy numbers than the rS27T-10 and rS27T-S297A-10 groups at 3–8 dpi, although these differences were also not significant (Fig. 8B). The viral titers (Fig. 8C and D) and DNA copy numbers from anal swabs exhibited similar trends.

For viral load assessment in the organs of infected raccoon dogs, animals inoculated with various viruses were euthanized at 8 dpi. Intestinal tissues were collected for viral DNA quantification via qPCR. Compared with the rRDPV-16 group, viral DNA copy numbers in the duodenum, colon, and cecum were significantly lower in the rT27S-A297S-16 group (Fig. 8E through G). Conversely, viral DNA copy numbers in the duodenum, colon, and cecum were significantly higher in the rS27T-10 and rS27T-S297A-10 groups compared with the rRDPV-10 group (Fig. 8H through J). No significant differences were observed in other tissues (Fig. S8).

### Structural analysis

**Fig 9:**
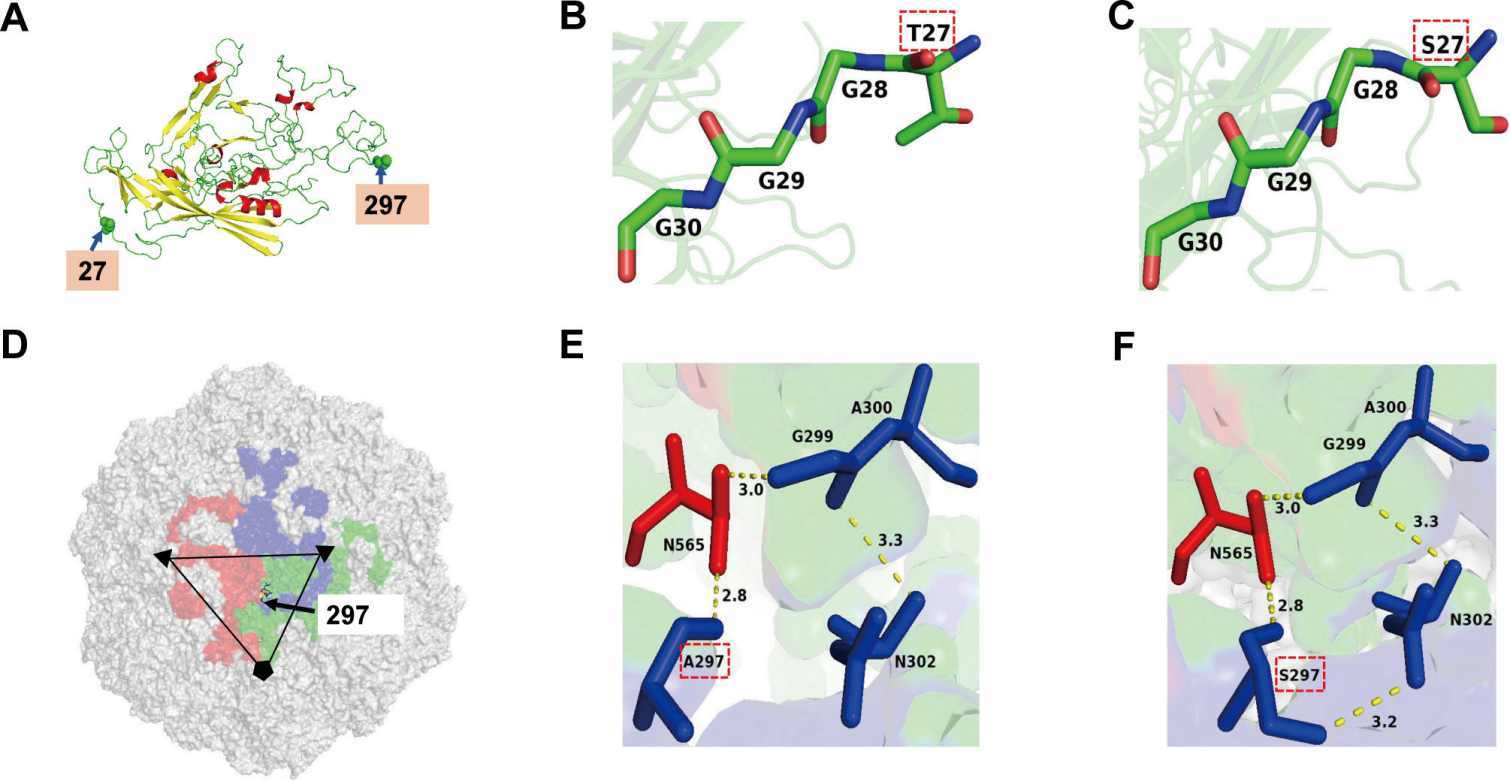
The structure of VP2 proteins. (**A**) The VP2 subunit was predicted using the Protein Data Bank (PDB) template 4 dpv. The 27 and 297 amino acid residues are located in loop regions, with residue 27 at the VP2 N-terminus. Helices are depicted in red, sheets in yellow, and loops in green. (**B and C**) Structural diagrams illustrate how the T27 or S27 mutations affect neighboring residues. (**D**) Icosahedral structure prediction was performed based on the Protein Data Bank (PDB) template with ID 1ijs. The 297 residue is located within the 3-fold spike receptor-binding region on the VP2 surface. (**E and F**) Structural diagrams highlight how the A297 or S297 mutations influence neighboring residues, emphasizing the formation of hydrogen bonds.

To determine whether mutations at residues 27 and 297 alter viral structural stability and thereby modulate receptor binding and pathogenicity. The three-dimensional structures of rRDPV-16 and rT27S-A297S-16 VP2 were predicted using SWISS-MODEL, with models built based on Protein Data Bank (PDB) identifiers 4dpv (Fig. 9A) and 1ijs (Fig. 9D), the latter representing the crystal structure of CPV VP2. Amino acids at positions 27 and 297 were analyzed using PyMOL. Approximately 87% of the N-terminus resides within the capsid, whereas about 13% extends outside (28). The N-terminus, rich in glycine residues near the 5-fold symmetry axis pores, is flexible and can adopt multiple conformations, enabling it to enter and exit the capsid through these pores (3). Position 27, located at the N-terminus of the VP2 subunit (Fig. 9A), plays a critical role in viral infection, potentially by exposing the N-terminus outside the capsid and thereby facilitating host cell binding (28, 29). The mutation of serine (S) to threonine (T) at position 27 induces a local alteration in the spatial conformation (Fig. 9B and C). Position 297, located in the loop region of VP2 involved in receptor binding, is near the surface spike determinant region at residue 300 in the icosahedral structure (Fig. 9D). The substitution of serine with alanine at position 297 disrupts hydrogen bonding, such as the interaction between N302 and A297, which may result in local conformational changes (Fig. 9E and F).

To investigate the effects of mutations at residues 27 and 297 on the interaction between RDTfR and the virus, we compared the sequence and glycosylation characteristics of TfRs from dogs, raccoon dogs, cats, and black-backed jackals (bbj) (Fig. S9). Sequence alignment revealed that RDTfR shares 98% identity with bbj TfR, and both lack an N-linked glycosylation site at residue 384, which has been shown to influence CPV binding (30). Therefore, the published cryo-EM structure of CPV in complex with bbj TfR was used as a structural model for RDPV–RDTfR docking (31) (31). H-dock–based docking and molecular dynamics simulations further suggested that the mutation at residue 297 may slightly alter the conformation of VP2 and reduce its binding affinity to RDTfR (Fig. S10 and S11).

## DISCUSSION

High-frequency mutations in the VP2 protein are key drivers of host adaptation and the evolutionary fitness of CPV (32–34). Previous studies have shown that amino acid substitutions at critical VP2 sites can markedly alter viral interactions with host TfRs, thereby promoting viral evolution (25, 35). Notably, the RDPV strain isolated in 2016 exhibited enhanced pathogenicity, resulting in more severe clinical signs and increased mortality in raccoon dogs (21, 36). Through systematic analysis of mutations at VP2 residues 27 and 297, we, for the first time, established their association with RDTfR binding capacity, viral replication, and pathogenicity: (i) rRDPV-16 showed significantly higher viral titers and DNA copy numbers than its mutant, whereas rRDPV-10 exhibited the opposite trend (ii); both rRDPV-16 and rS27T-S297A-10 displayed stronger binding affinity to RDTfR; and (iii) compared with their parental viruses, rRDPV-16 and rS27T-S297A-10 replicated more efficiently and caused more severe disease in raccoon dogs.

Mutations at key amino acid residues in VP2 have been shown to regulate host adaptation and infectivity of CPV. For instance, the E298A, T389A, and K387A/T389A substitutions render CPV nonviable in A72 or NLFK cells (37), whereas FPV-specific mutations, such as 93K, 103V, or 323N, markedly impair viral replication in A72 cells (38). In this study, we found that the S27T/S297A double mutation in RDPV VP2 significantly enhanced viral replication in F81 and CRFK cells (Fig. 2; Fig. S3), suggesting a potential increase in *in vivo* transmissibility. Notably, under identical infection conditions (MOI = 0.01), CPV consistently exhibited higher viral titers in F81 cells than in CRFK cells, which correlated with more prominent cytopathic effects observed in F81 cells. This may reflect differential cellular support for viral replication across cell lines and helps explain why F81 cells are widely used for CPV propagation and study (11, 39).

The RDPV VP2 protein mediates cellular entry by binding to host TfRs, followed by clathrin-mediated endocytosis (40–43). Mutations at VP2 residues 299 and 300 have been shown to alter capsid conformation, thereby affecting the virus’s ability to bind TfRs from different host species (5, 44). In this study, we found that residue 297 is structurally adjacent to position 300, and its substitution may indirectly influence TfR binding through local conformational changes (28). Notably, the S297A mutation exhibited signatures of positive selection, suggesting a potential role in host adaptation (22–24). Residue 27, located on the inner surface of the VP2 N-terminus, may become transiently exposed during receptor-induced conformational shifts (20, 45). However, the precise functional mechanisms of these mutations remain to be experimentally validated.

Structural modeling predicted that the A297S mutation forms a novel internal hydrogen bond network within VP2 (e.g., between S297 and N302) (Fig. 9), which may influence receptor binding. This observation is consistent with previous findings that mutations at VP2 residues 93 and 323 in CPV alter its affinity for canine TfR by modulating capsid conformation (46). Experimental data further demonstrated that the S27T/S297A double mutation significantly enhanced RDPV binding to RDTfR, as consistently confirmed by binding assays in F81, 293T, and TRVb cells, as well as by ELISA and BLI analyses. Both single mutations at positions 27 and 297 also reduced receptor binding, although to varying degrees. Virus-receptor binding can occur through multiple mechanisms, as exemplified by the various binding modes between Eastern equine encephalitis virus and very low-density lipoprotein receptor (47). Our BLI results show that compared with the 2016 single-mutant strain, the rT27S-A297S-16 variant exhibits a smaller reduction in affinity for RDTfR, suggesting that the binding modes may differ between these variants. Notably, rS27T-S297A-10 bound RDTfR more strongly than rRDPV-16, implying potential synergistic effects of other residues. Previous studies have shown that CPV can interact with human TfR (48). In our study, the rS27T-10 mutant exhibited binding to RDTfR in HEK 293T cells comparable with that of the parental strain. This observation may be attributed to interference from endogenous human TfR expressed in these cells. Collectively, these results confirm the critical regulatory roles of mutations at positions 27 and 297 in modulating viral receptor binding.

Although previous studies have largely focused on evaluating mutation effects *in vitro*, our investigation reveals the *in vivo* pathogenic consequences of mutant strains in raccoon dogs. Compared with the parental virus, both rRDPV-16 and rS27T-S297A-10 exhibited enhanced replication and increased virulence *in vivo*, findings that are broadly consistent with their higher receptor-binding affinities observed *in vitro*. These results support a positive association between the S27T/S297A mutations, TfR binding, and pathogenicity. However, an exception was observed. Although rS27T-S297A-10 demonstrated greater TfR affinity and replication capacity (Fig. 4H and 8), its pathogenicity was lower than that of rRDPV-2016 (Fig. 5 and 6). This discrepancy may stem from synergistic effects involving other critical residues, such as positions 80, 93, 103, and 323 (Fig. S1) (44, 49, 50). It is worth noting that high receptor affinity does not always equate to increased infectivity. For instance, CPV binds canine TfR with relatively low affinity but nonetheless spreads efficiently (31), likely due to the presence of an N-linked glycosylation site at position 384 in canine TfR, which has limited impact on CPV infection but strongly inhibits FPV binding and infection (6). Taken together, our results confirm a general positive correlation between receptor affinity and pathogenicity; however, this relationship may be influenced by additional viral or host factors and constrained by the use of species-specific animal models.

Our study confirms that rRDPV-16 exhibits stronger replication capacity, virulence, and RDTfR binding affinity compared with rT27S-A297S-16. The synergistic effects of the S27T and S297A mutations in the VP2 protein enhance its interaction with RDTfR, thereby significantly improving viral replication efficiency. These findings not only elucidate the molecular basis of RDPV pathogenicity but also underscore the importance of continuous monitoring of VP2 mutations for the early detection of highly virulent strains.

## MATERIALS AND METHODS

### Cells and viruses

Infectious viruses were propagated in feline kidney (F81) cells, which were cultured in minimum essential medium (MEM) (Gibco) supplemented with 10% fetal bovine serum (FBS) at 37°C in a humidified incubator with 5% CO2. Human embryonic kidney (HEK 293T) and feline kidney cells (CRFK) were maintained in Dulbecco’s modified Eagle’s medium (DMEM) (Gibco) with 10% FBS under the same incubation conditions. The TRVb cell line, derived from Chinese hamster ovary (CHO) cells and lacking endogenous transferrin receptor (TfR) expression, was maintained in F-12 Nutrient Mixture (Kaighn’s modification) (Gibco) with 10% FBS at 37°C in a humidified incubator with 5% CO₂. The TRVb cell line was generated and screened by GenScript Biotech Corporation (Nanjing, China), as documented in Supplementary Information. Spodoptera frugiperda (Sf9) cells were cultured in Sf-900™ II SFM (Gibco) with 10% FBS at 27°C in a humidified incubator.

The RDPV/CL/2016 and RDPV/FLD/2010 strains, isolated from infected animals across various provinces in China, are routinely maintained in our laboratory (RDPV/CL/2016 GenBank: PQ807006; RDPV/FLD/2010 GenBank: PQ807007).

### Genetic evolution of RDPV sequences

VP2 sequences from dogs and raccoon dogs were retrieved from the NCBI database (https://www.ncbi.nlm.nih.gov/nucleotide/). A phylogenetic tree was generated using distance-based maximum likelihood analysis in MEGA 6.0, with bootstrap values determined from 1,000 replicates.

### Construction of cDNA clones and expression plasmids

cDNA clones of rRDPV-16 and rRDPV-10 were generated following the protocol outlined by Yuan et al. (51). Site-directed mutagenesis of residues 27 and 297 in the VP2 protein of the RDPV/CL/2016 and RDPV/FLD/2010 strains was conducted using a Q5 site-directed mutagenesis kit (New England Biolabs). In addition, 5 µg of each rRDPV-16 and rRDPV-2010 plasmid was transfected into F81 cells using Lipofectamine 3000 transfection reagent (Invitrogen), according to the manufacturer’s instructions. After 48 h post-transfection, the supernatants were transferred to fresh F81 cells and passaged until the cytopathic effect (CPE) stabilized. The viruses were passaged for over three generations, collected through repeated freeze-thaw cycles, and titrated.

For the construction of eukaryotic expression plasmids for raccoon dog ectodomain transferrin receptor (RDTfR), the ectodomain of RDTfR (XM_055316305), starting at amino acid residue 121, was cloned into the pFastBacI vector, along with the N-terminal signal peptide GP67 and the C-terminal 6× His tag, generating the pFastBacI-RDTfR plasmid. To generate the pCDNA3.4-RDTfR plasmid, RDTfR and a C-terminal HA tag were inserted into the pCDNA3.4 vector, which contained the BspEI and EcoRV restriction sites.

### Virus rescue confirmation

To verify successful virus rescue, F81 cells were infected with various strains (rRDPV-16, rT27S-16, rA297S-16, rT27S-A297S-16, rRDPV-10, rS27T-10, and rS27T-S297A-10) and incubated at 37°C for 36 h. Later, the cells were washed three times with cold PBST and fixed with a cold formaldehyde-acetone solution for 15 min at room temperature. The cells were then incubated with the 6A8 mouse anti-VP2 antibody for 1 h at 37°C (52). After washing, the cells were treated with a fluorescein isothiocyanate (FITC)-conjugated goat anti-mouse IgG secondary antibody for 1 h at 37°C. After three additional washes with PBST, the F81 cells were examined under a fluorescence microscope (LEICA DMI3000 B, Germany).

### Viral growth kinetics

F81 and CRFK cells were infected with RDPV at multiplicities of infection (MOI) of 0.01. Cell supernatants were collected at 24, 48, 72, and 96 h post-infection (hpi). These samples were serially diluted and subsequently added to F81 cells in 96-well plates, with eight replicates per 10-fold dilution. The plates were incubated at 37°C for 5 days to monitor cytopathic effects and determine viral titers using the Reed and Muench method.

### Viral DNA extraction and quantification

Viral DNA was extracted using an automated nucleic acid extraction system (TIANLONE, China). Viral loads were quantified by real-time PCR using the RealStar Fast SYBR qPCR Mix kit (A301-05; GenStar, Chang Chun, China). PCR amplification was performed on a fluorescent qPCR instrument (LightCycler 96, Roche, Switzerland/USA) following the manufacturer’s instructions. The VP2 gene fragment of RDPV was targeted for amplification in the real-time PCR assays. A standard VP2 sample was amplified using the following primers: VP2 forward, 5´-GAAATCCCCACACCACCAGAA-3´ and VP2 reverse, 5´-CGGTGGTCAGCCTGCTGTCAGAAAT-3´. Viral load quantification was performed using the standard curve method.

### Virus purification and characterization

F81 cells in 75 cm² culture flasks were infected with various strains (rRDPV-16, rT27S-16, rA297S-16, rT27S-A297S-16, rRDPV-10, rS27T-10, rS297A-10, or rS27T-S297A-10) at a multiplicity of infection (MOI) of 0.01. After 3 days, the cells and culture supernatants underwent two freeze-thaw cycles, followed by centrifugation and filtration through a 0.22 µm membrane filter for clarification. Virus purification was performed using a PrePack Purrose Shell V15 column (ZA76154-02, Qianchun Bio) and analyzed using an AKTA Pure system (GE, Sweden). VP2 protein was detected by western blotting using the mouse monoclonal antibody 6A8.

### Expression and purification of RDTfR

The recombinant pFastBacI-RDTfR plasmid was transfected into Sf9 cells using Lipofectamine 3000 (Invitrogen) according to the manufacturer’s instructions. The transfected cells were cultured and collected for analysis of TfR expression using 10% SDS-PAGE and western blotting. A mouse monoclonal antibody (Servicebio) was employed to detect His-tagged TfR fusion proteins.

RDTfR was purified by affinity chromatography using ProteinIso® Ni-NTA Resin (DP101-01, TransGen Biotech, Chang Chun, China) and eluted with 100 mM imidazole according to the manufacturer’s protocol. The TfR-containing fractions were dialyzed overnight in PBS. Protein concentrations were quantified using a bicinchoninic acid (BCA) protein assay kit (Beyotime). The purity of the purified TfR was assessed by SDS-PAGE, and VP2 was detected by western blotting using the mouse monoclonal antibody 6A8.

### Western blot analysis of RDTfR and virus

RDTfR and the virus were separated by SDS-10% polyacrylamide gel electrophoresis (SDS-PAGE) and transferred to nitrocellulose membranes. The membranes were then blocked and incubated with the primary antibody at the appropriate dilution in phosphate-buffered saline (PBS) containing 3% (wt/vol) skim milk. Mouse monoclonal antibody 6A8 was used to detect VP2 expression. Western blotting was performed using an HRP-conjugated anti-mouse IgG secondary antibody, following the manufacturer’s instructions (BBI). Immunoreactive bands were visualized using an ECL detection reagent (BOSTER), and images were captured.

### Virus attachment assay

F81 cells were seeded in 12-well plates and incubated overnight. The next day, the cells were fixed with 4% paraformaldehyde at 4°C for 30 min. Purified virus (1  mL at 40  µg/mL) was then added to each well, and virus adsorption was carried out at 4°C for 2 h. After incubation, the cells were washed three times with PBS to remove unbound virus, and viral DNA was quantified by qPCR.

To evaluate the attachment of virus particles to F81 cells, indirect immunofluorescence was performed on fixed, non-permeabilized cells. Cells were incubated with a mouse monoclonal antibody against VP2, followed by a FITC-conjugated goat anti-mouse IgG secondary antibody. Fluorescence signals were examined using a fluorescence microscope (LEICA DMI3000 B, Germany).

### Binding of RDPV to RDTfR expressed in HEK 293T and TRVb cells

HEK 293T and TRVb cells were transfected with the pCDNA3.4-RDTfR plasmid and incubated for 24 h. After transfection, the cells were fixed with 4% paraformaldehyde at 4°C for 30 min, followed by incubation with virus at 4°C for 2 h. Cells were then washed with PBS to remove unbound virus, and DNA was extracted for quantification of viral DNA by qPCR.

Indirect immunofluorescence was performed to assess the expression of RDTfR-HA and virus binding. After fixation, the cells were permeabilized at room temperature with 0.1% Triton X-100 for 10 minutes. RDTfR-HA expression was detected using rabbit anti-HA antibody as the primary antibody, and Alexa Fluor 594-labeled goat anti-rabbit IgG as the secondary antibody. To assess virus binding to RDTfR, non-permeabilized fixed cells were incubated with mouse anti-VP2 monoclonal antibody as the primary antibody and FITC-conjugated goat anti-mouse IgG as the secondary antibody. Cells were subsequently analyzed using fluorescence microscopy (LEICA DMI3000 B, Germany).

### ELISA for detection of RDTfR binding

ELISA plates were coated overnight at 4°C with purified viral particles (rRDPV-16, rT27S-16, rA297S-16, rT27S-A297S-16, rRDPV-10, rS27T-10, rS297A-10, or rS27T-S297A-10) at a concentration of 200 µg/mL. The plates were then blocked with 5% milk in PBST. RDTfR was diluted and added to the plates, followed by incubation overnight. After washing, His-tagged mouse monoclonal antibodies (Servicebio) were applied. The plates were subsequently incubated with HRP-conjugated anti-mouse IgG (BBI). Absorbance at 450 nm was measured after color development.

### Biolayer interferometry (BLI)

The virus was immobilized on an APS probe (20-5056, Gator Bio, Silicon Valley, USA) using Gator Prime (Gator Bio, Silicon Valley, USA) and phosphate-buffered saline (PBS) as the running buffer. Various concentrations of RDTfR in PBST (PBS containing 0.05% Tween-20) were then passed over the APS probe. The resulting data were analyzed using Gator Evaluation Software (Gator Prime, Silicon Valley, USA) and fitted to a 1:1 binding model.

### *In vivo* infection assay

Groups of 2-month-old raccoon dogs were inoculated with 10^5.0^ TCID_50_ of rRDPV-16, rT27S-16, rA297S-16, rT27S-A297S-16, rRDPV-10, rS27T-10, or rS27T-S297A-10 per raccoon dog. The infected animals were monitored daily over an 8-day period for survival, clinical symptoms, and the presence of diarrhea. Symptom severity was graded on a scale from 0 to 4, where 0 indicated healthy animals, 1 represented mild symptoms, 2 indicated moderate symptoms, 3 denoted severe symptoms, and 4 was assigned to deceased animals.

To evaluate the pathogenicity of the virus, groups of 2-month-old raccoon dogs were infected with 10^5.0^ TCID_50_ of rRDPV-16, rT27S-16, rA297S-16, rT27S-A297S-16, rRDPV-10, rS27T-10, or rS27T-S297A-10 per animal. Clinical symptoms, diarrhea, rectal swabs, and body temperature were monitored daily. Clinical symptoms were scored based on the severity of lethargy, appetite, ocular discharge, and vomiting. The scores were as follows: 0, no symptoms; 1, depression and ocular discharge; 2, poor appetite, lethargy, and vomiting; 3, no appetite, lethargy with inability to rise, sunken eyes, and ocular discharge; and 4, death. After 8 days, the raccoon dogs were euthanized, and both small and large intestines were harvested, weighed, and homogenized in 1 mL of PBS. After high-speed centrifugation to clarify the samples, viral DNA copies were quantified via qPCR on the resulting supernatants.

For histopathological analysis, groups of 2-month-old raccoon dogs were either administered MEM (control group) or infected with viral strains. After 8 days, the animals were euthanized, and the small and large intestines were collected and fixed in 4% paraformaldehyde at room temperature. Following fixation, the samples underwent hematoxylin and eosin (H&E) staining (Servicebio). The stained sections were examined under a light microscope, and images were captured for further analysis.

### Structural prediction and analysis of VP2 and RDTfR

To examine the spatial positioning of the two consistently identified amino acid mutations within VP2, we utilized SWISS-MODEL (https://swissmodel.expasy.org/) to predict the protein’s three-dimensional structure. Structural analysis was performed using PyMOL (53, 54). Furthermore, the three-dimensional structure of RDTfR was predicted and compared with the sequence of TfRs from dogs, cats, and black-backed jackals.

### Docking models and molecular dynamics simulations

The initial docking models for rRDPV-16-VP2 (27T, 297A), rT27S-A297S-16-VP2 (27S, 297S), and RDTfR were generated using the Hdock server (http://hdock.phys.hust.edu.cn) (55). The top-scoring model was refined using ITScorePP and further optimized through 100 ns molecular dynamics (MD) simulations conducted with GROMACS 2023.2 (https://www.gromacs.org) (56). Simulations employed the amber99sb-ildn force field, with small-molecule parameters generated by Antechamber (AM1-BCC charges). The protein–ligand complex was solvated in a TIP3P water box, using 1.0 nm cutoffs for electrostatic and van der Waals interactions and PME for long-range electrostatics. After energy minimization, the system underwent 200 ps NVT and 100 ps NPT equilibration at 303.15 K and 1 bar, using V-rescale and Parrinello–Rahman methods, respectively. Structural stability and dynamics were assessed via RMSD and radius of gyration, whereas interaction energies were calculated with the mdrun module. Protein-protein interaction interfaces were analyzed using PyMOL and the Molecular Operating Environment (MOE) (53, 54). The binding-free energies between RDTfR and rRDPV-16-VP2 (27T, 297A) or rT27S-A297S-16-VP2 (27S, 297S) were calculated from molecular dynamics simulations in GROMACS based on the final docking model.

### Statistical analysis

Statistical analysis was performed using GraphPad Prism 9 (GraphPad Software, San Diego, CA, USA). Variations between the groups were evaluated using analysis of variance (ANOVA). Statistical significance levels: *P < 0.05; **P < 0.01; ***P < 0.001; ****P < 0.0001; ns, not significant.

## Data Availability

The data sets used and analyzed in this study are available from the corresponding author upon reasonable request. The sequences of RDPV/CL/2016 and RDPV/FLD/2010 have been deposited in GenBank with the accession numbers PQ807006 and PQ807007, respectively.

## SUPPLEMENTAL MATERIAL

Supplemental figures jvi.01012-25-s0001.docx Figures S1 to S11.

Supplemental text jvi.01012-25-s0002.docx Report on genomic editing of TRVb cells via GenCRISPR-mediated engineering.

ASM does not own the copyrights to Supplemental Material that may be linked to, or accessed through, an article. The authors have granted ASM a non-exclusive, world-wide license to publish the Supplemental Material files. Please contact the corresponding author directly for reuse.
