# Supplementary material for: Residues 27T and 297A in VP2 contribute to the enhanced replication and pathogenicity of raccoon dog parvovirus
Source: J Virol. 2025 Sep 5;99(10):e01012-25. doi: 10.1128/jvi.01012-25 (PMC12548415; doi:10.1128/jvi.01012-25)
Supplement: Supplemental figures — Figures S1 to S11. [file jvi.01012-25-s0001.docx]

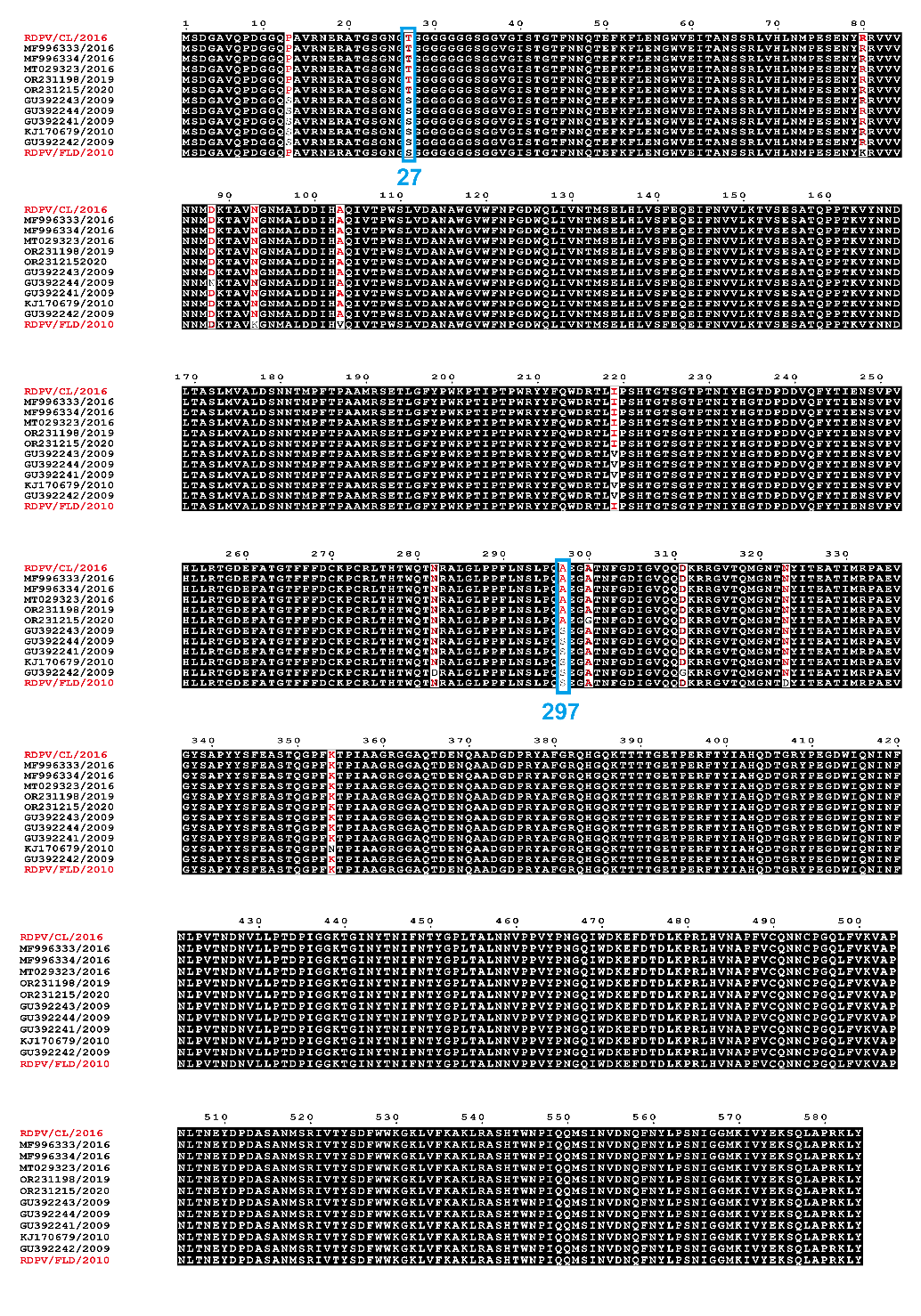


**Fig. S1 VP2 sequence alignment of representative RDPV/CL/2016 and RDPV/FLD/2010 Strains.** Amino acid positions are based on the RDPV/CL/2016 VP2 sequence. The mutated amino acids at positions 27 and 297 in VP2 are indicated by

cyan boxes. Variable amino acid residues are shown with a white background and were distinguished using black and red fonts, whereas fully conserved residues were highlighted in black.


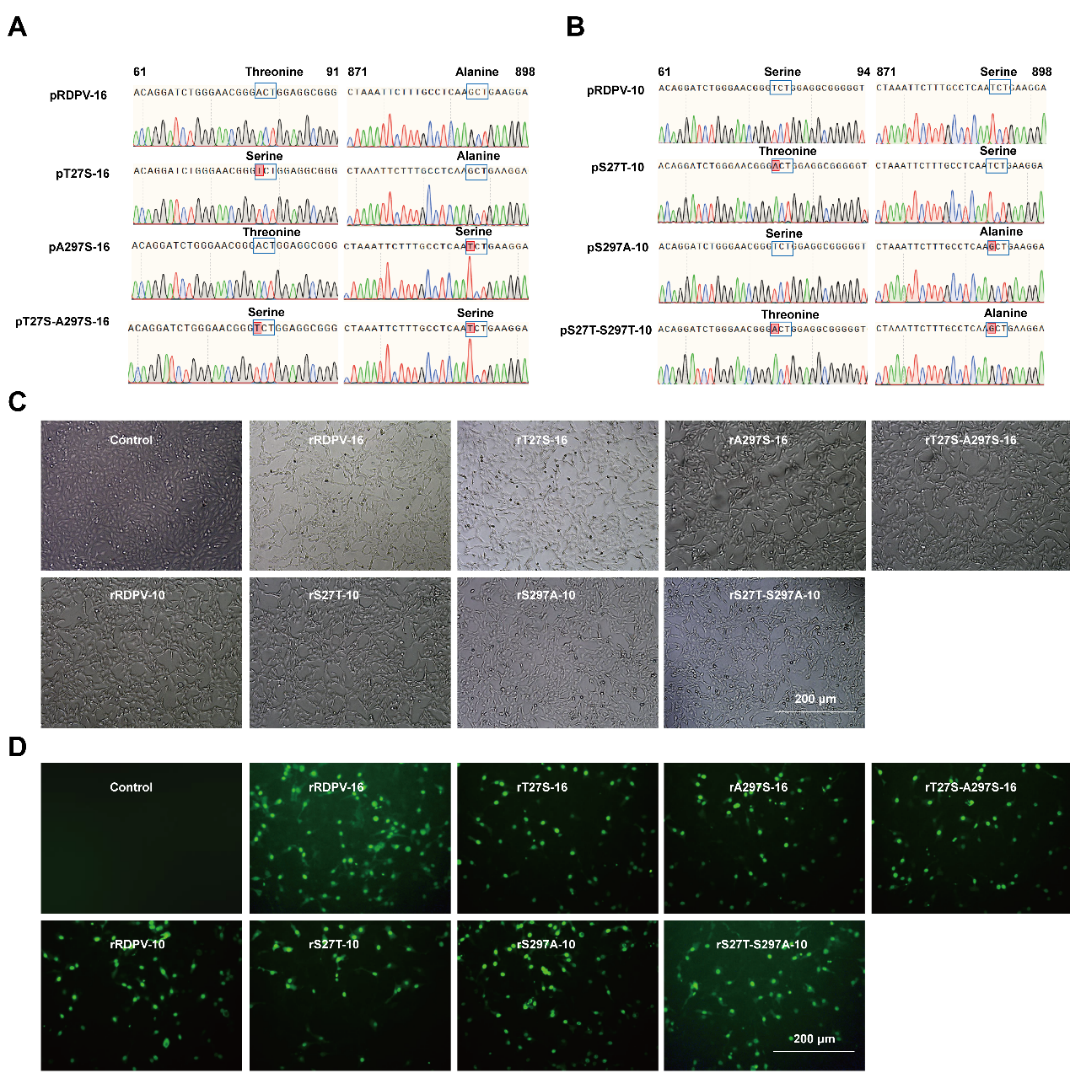


**Fig. S2 Sequencing of rescued virus cDNA plasmids and identification of recombinant mutant viruses.** (A) Sequencing results of the pRDPV-16 plasmid and its mutants, including pT27S-16, pA297S-16, and pT27S-A297S-16. (B) Sequencing results of the pRDPV-10 plasmid and its mutants, including pS27T-10, pS297A-10, and pS27T-S297A-10. Plasmids were verified using the Sanger sequencing method (Sangon). (C) Cytopathic effect and (D) Immunofluorescence assay. F81 cells were infected with third-generation recombinant viruses (rRDPV-16, rRDPV-10, and the mutant virus) for 36 hours, and the cells were then analyzed under a microscope. Scale bar: 200 μm.


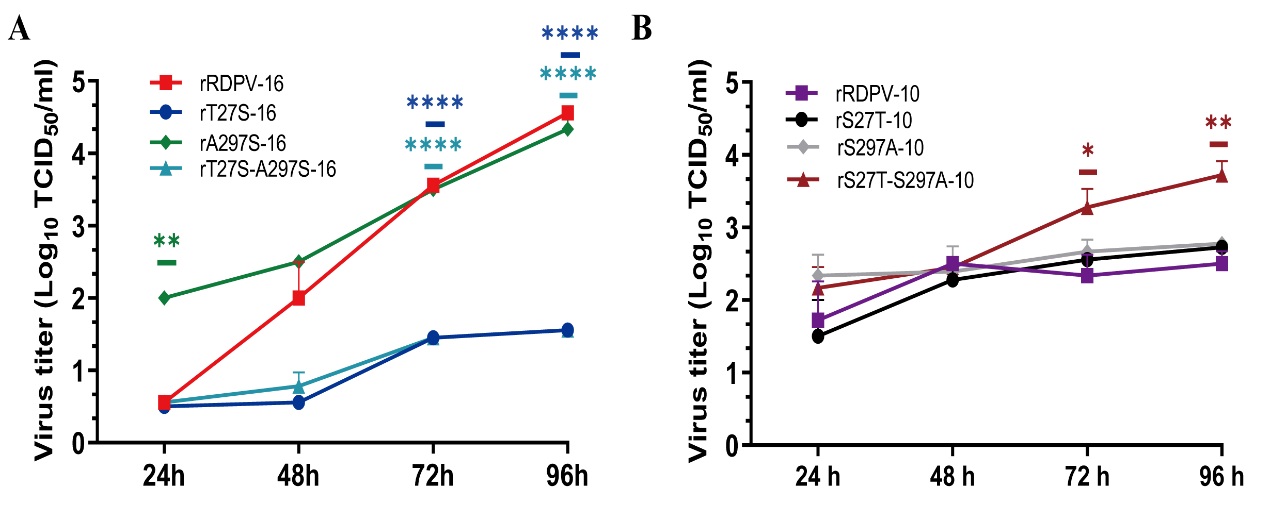


**Fig. S3 Growth kinetics of rescued viruses with amino acid mutations in CRFK cell.** (A and B) Reverse cDNA versions of rRDPV-16 and rRDPV-10 viruses containing rT27S-16, rA297S-16, rT27S-A297S-16, rS27T-10, rS297A-10, and rS27T-S297A-10, were used to infect CRFK cells at an MOI of 0.01, which were collected and quantified using a TCID_50_ assay at 24, 48, 72, and 96 hpi.


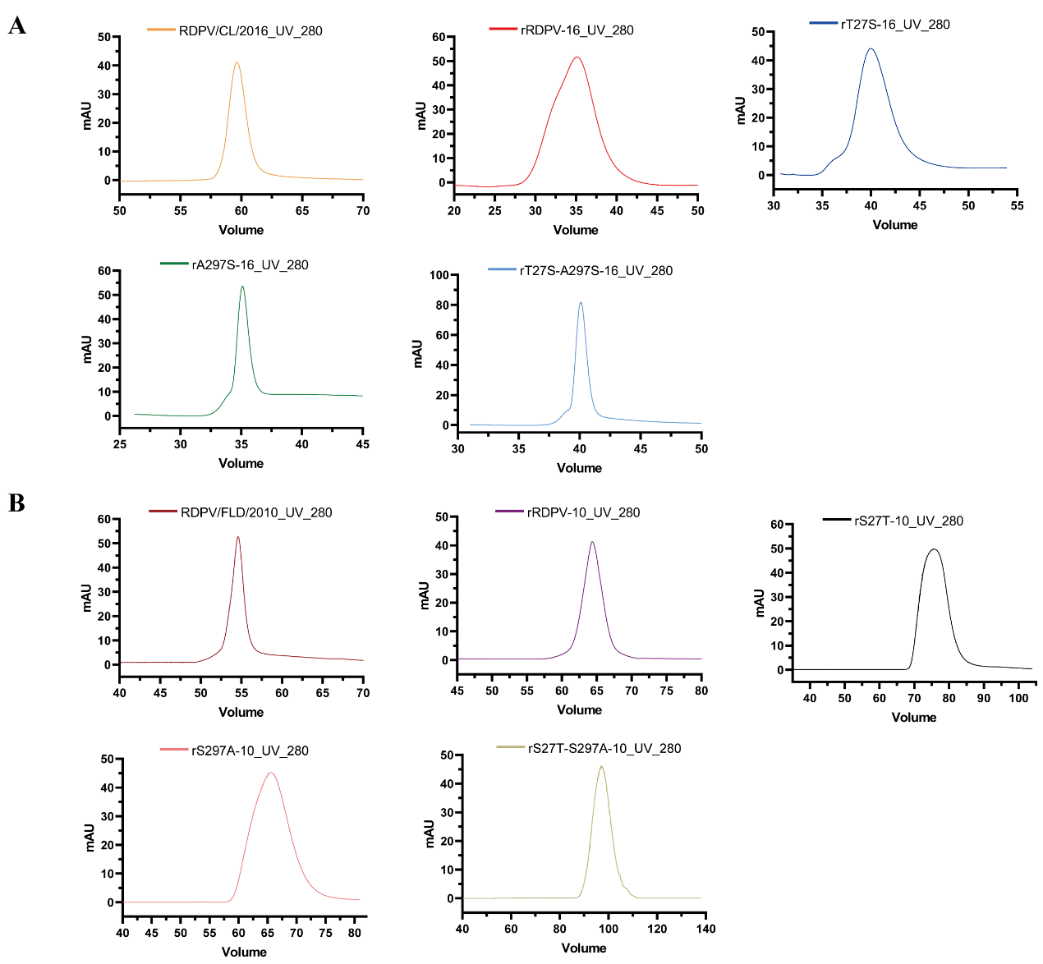


**Fig. S4 Virus purification.** (A) UV280 values of RDPV/CL/2016, rRDPV-16, rT27S-16, rA297S-16 and rT27S-A297S-16 using PrePack Purrose Shell V15 column from AKTA Purifier. (B) UV280 values of RDPV/FLD/2010, rRDPV-10, rS27T-10, rS297A-10 and rS27T-S297A-16 using PrePack Purrose Shell V15 column from AKTA Purifier.


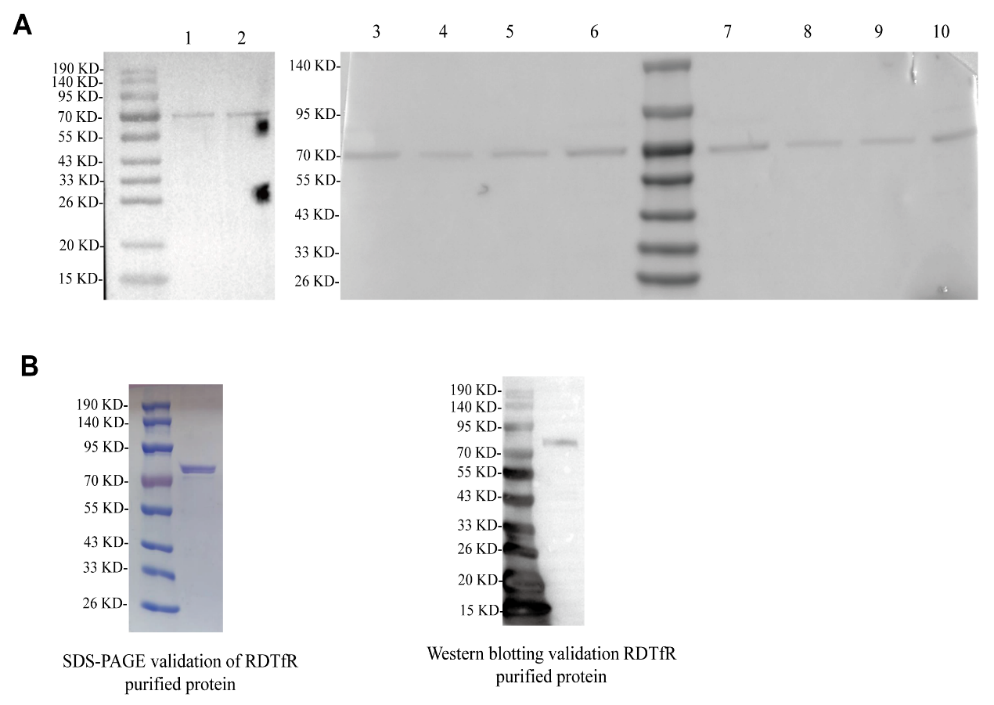


**Fig. S5 Validation of purified virus and RDTfR.** (A) Numbers 1 to 10 were RDPV/CL/2016, RDPV/FLD/2010, rRDPV-16, rT27S-16, rA297S-16, rT27S-A297S-16, rRDPV-10, rS27T-10, rS297A-10 and rS27T-S297A-10 purified viruses, respectively, identified by Western blotting using mouse VP2 antibody. The VP2 protein is approximately 70 kD in size. (B) Purified RDTfRs were confirmed through SDS-PAGE and validated with Western blotting using mouse His antibodies. The RDTfR protein is approximately 80 kD in size.


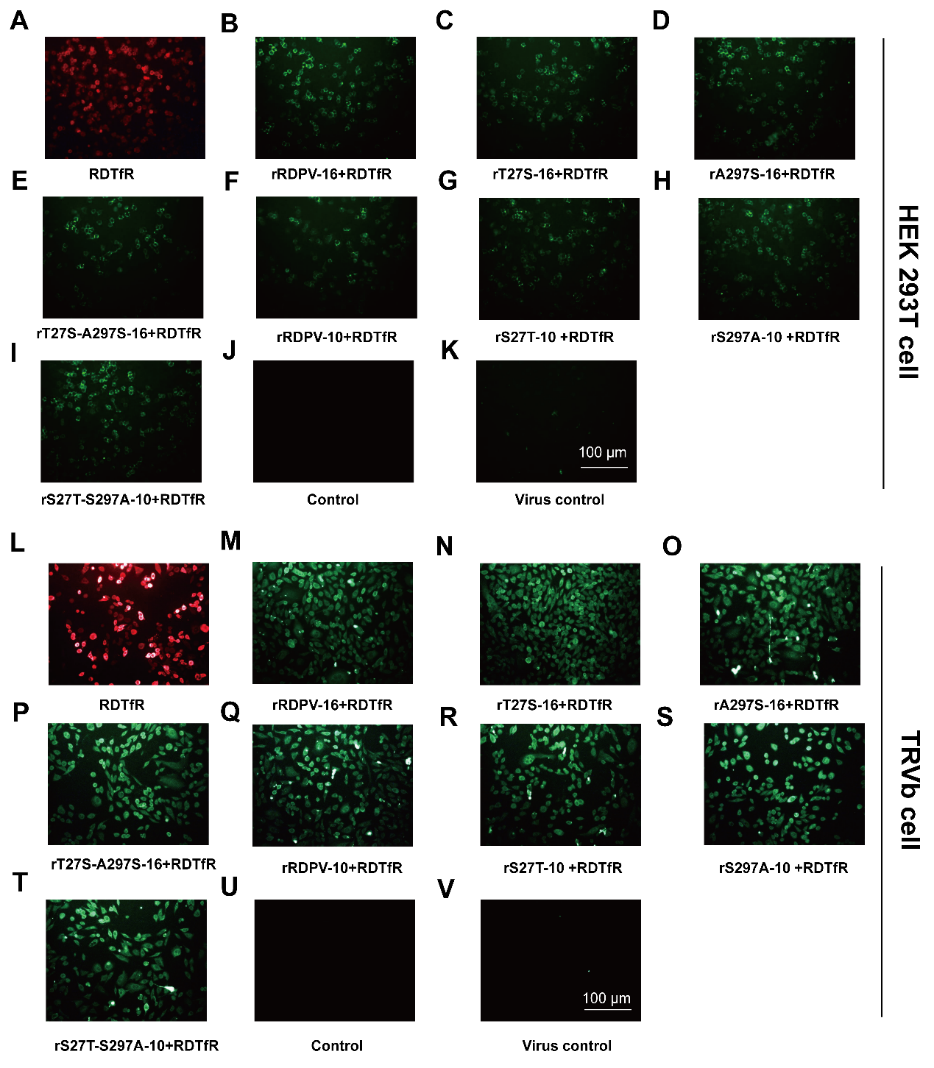


**Fig. S6 Immunofluorescence assay for identification of viral receptor binding.** (A and L) After 24 hours of transfection with the pCDNA3.4-RDTfR plasmid into HEK 293T or TRVb cells, RDTfR was detected using a rabbit anti-HA tag monoclonal antibody. The secondary antibody used was an Alexa Fluor 594-labeled goat anti-rabbit IgG. (B to I and M to T) After 24 hours of transfection with the pCDNA3.4-RDTfR plasmid into HEK 293T or TRVb cells, the virus was incubated at 4°C for 2 hours, and viral VP2 was identified using a mouse-VP2 6A8 monoclonal antibody. The secondary antibody was FITC-conjugated goat anti-mouse IgG. (J and U) HEK 293T or TRVb cells served as a cell control and were stained with rabbit anti-HA tag and mouse-VP2 6A8 monoclonal antibodies. (K and V) Virus control: HEK 293T or TRVb cells were inoculated with the virus at 4°C for 2 hours without transfection of the pCDNA3.4-RDTfR plasmid, followed by identification of VP2 using the mouse 6A8 monoclonal antibody. Both secondary antibodies were FITC-conjugated goat anti-mouse IgG antibodies. Cells were analyzed under a microscope. Scale bar: 100 μm.


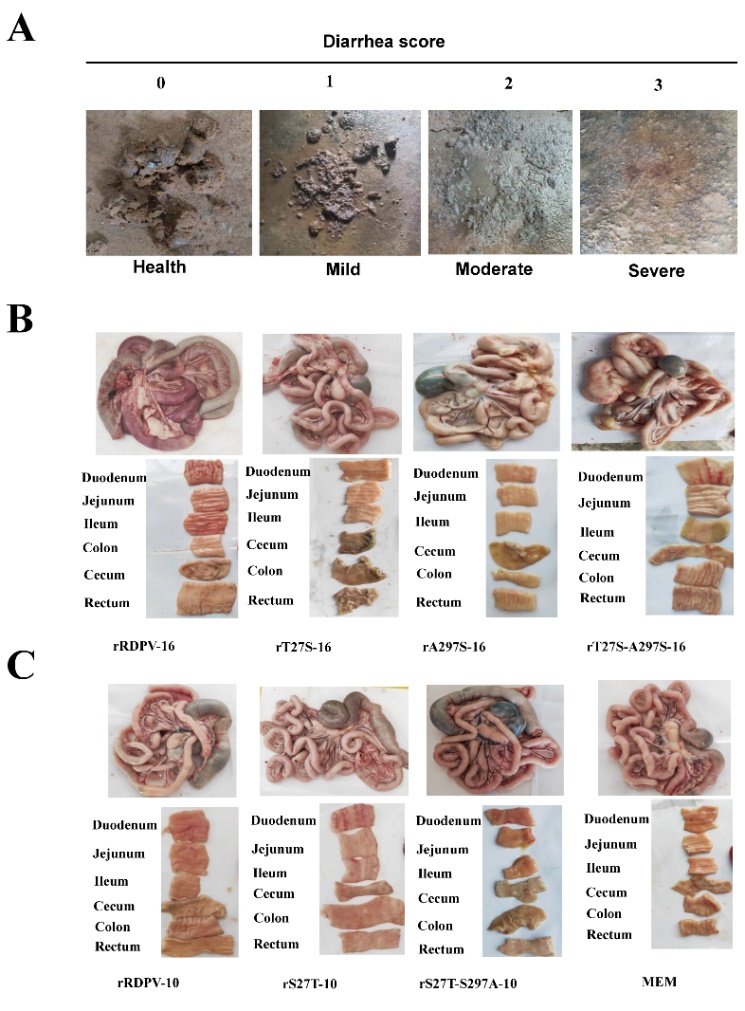


**Fig. S7 Intestinal macroscopic lesions.** (A) The diarrhea score of 0-3 corresponds to the stool consistency. (B and C) Macroscopic lesions in the intestine of raccoon dog infected with virus and uninfected controls.


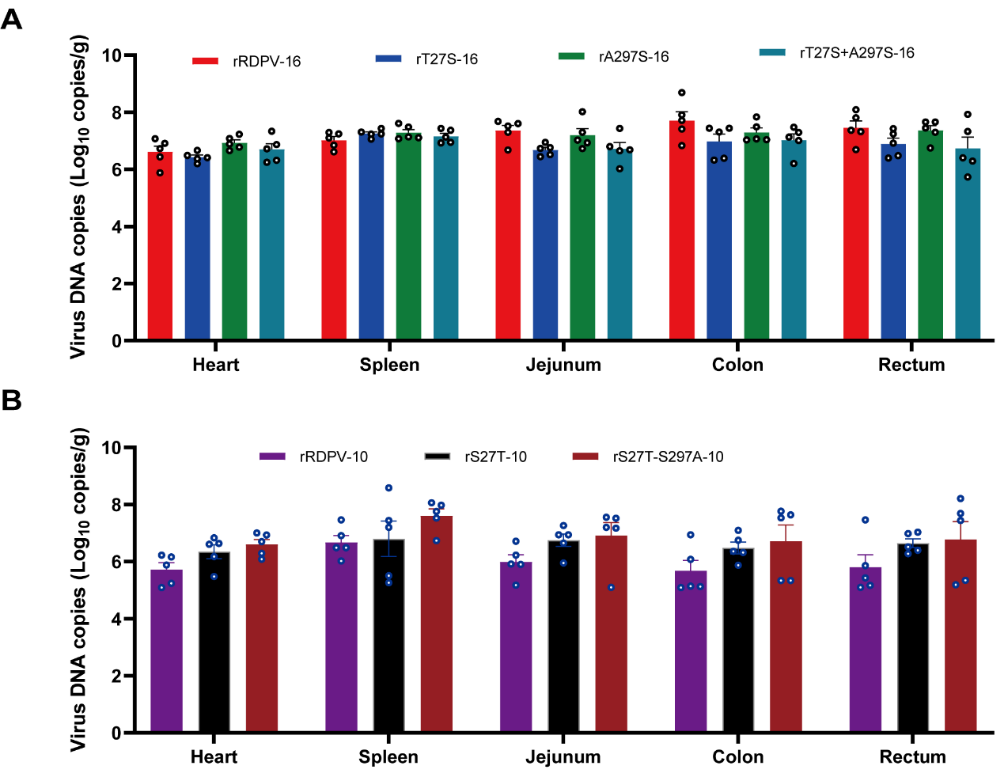


**Fig. S8 Tissue virus load measurement.** Heart, spleen, jejunum, colon, and rectum DNA copies number of raccoon dogs inoculated with RDPV.


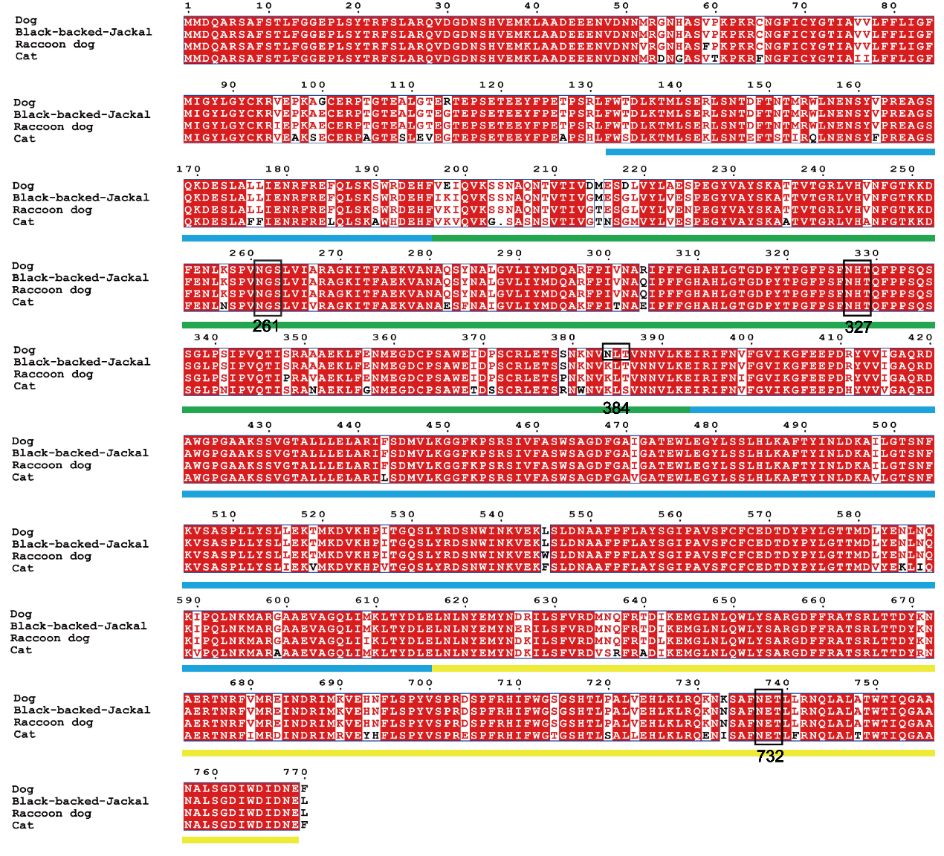


**Fig. S9 Aligned sequences of the dog, black-backed-jackal, raccoon dog, and cat TfRs.** Sequence variations between the dog, black-backed jackal, raccoon dog, and cat TfRs are highlighted with white shading. The three-dimensional structure of RDTfR was predicted using SWISS-MODEL, based on the PDB identifier 1cx8. The receptor ectodomain regions identified from the dog TfR structure are underlined in blue (protease-like domain), green (apical domain), and yellow (helical domain). Glycosylation sites are indicated by black boxes.


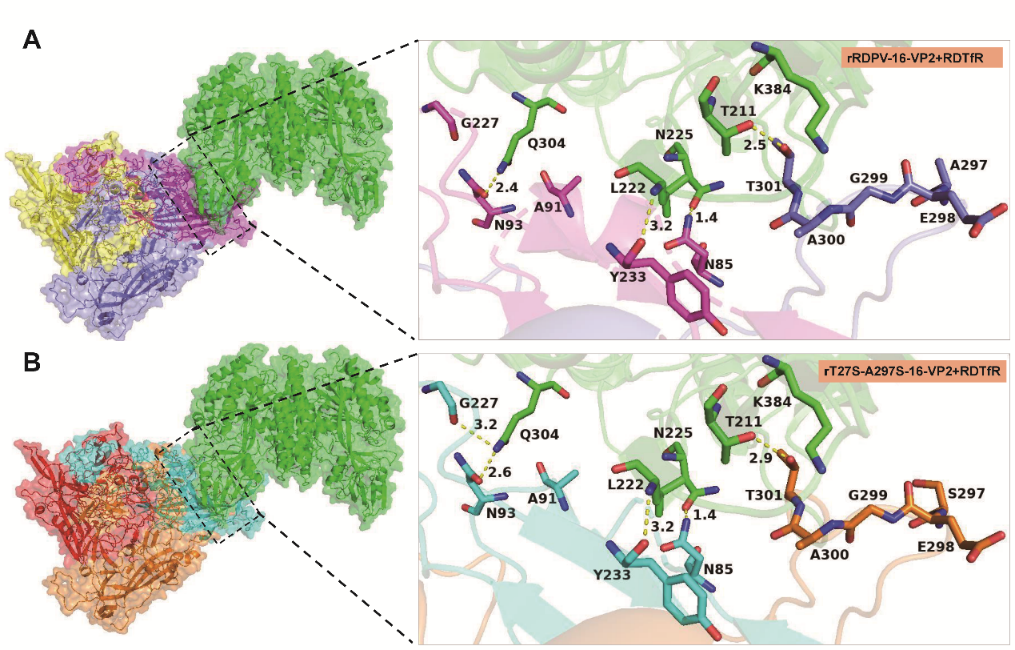


**Fig.S10** **Molecular docking of VP2 and RDTfR.** (A and B) The interactions between rRDPV-16-VP2 (27T, 297A) or rT27S-A297S-16-VP2 (27S, 297S) and RDTfR were analyzed using PyMOL and the Molecular Operating Environment (MOE). The trimer structure of rRDPV-16-VP2 (27T, 297A) is shown in yellow, blue, and pink, the trimer structure of rT27S-A297S-16-VP2 (27S, 297S) is shown in cyan, orange, red and RDTfR is depicted in green. Yellow dashed lines represent hydrogen bonds between residues of different subunits.


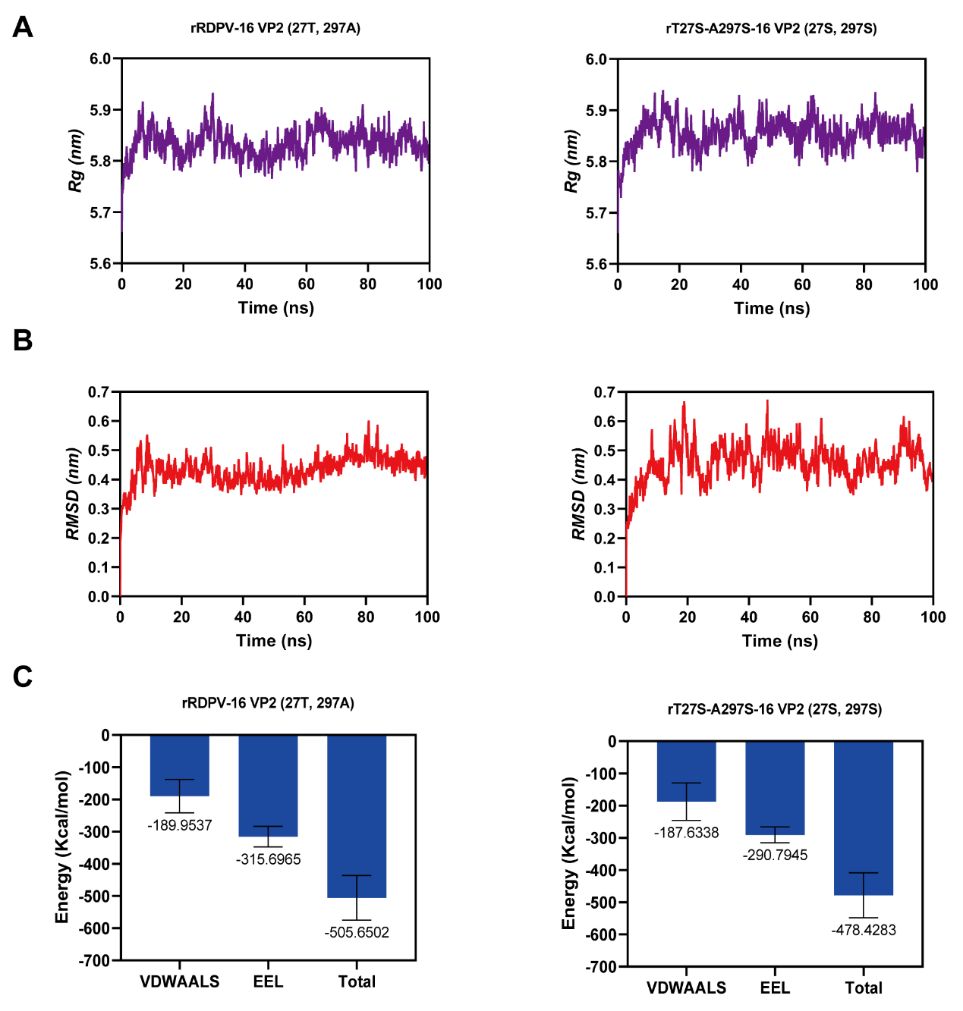


**Fig. S11 Binding free energy calculated for the VP2 and RDTfR proteins.** (A) Rg of the backbone atoms for the rRDPV-16-VP2-RDTfR complex (left) and the rT27S-A297S-16-VP2-RDTfR complex (right). (B) RMSD of the backbone atoms for the rRDPV-16-VP2-RDTfR complex (left) and the rT27S-A297S-16-VP2-RDTfR complex (right). (C) Binding free energy of the rRDPV-16-VP2 with RDTfR (left) and the rT27S-A297S-16-VP2 with RDTfR (right). Rg, radius of gyration; RMSD, root mean square deviation; RDTfR, raccoon dog transferrin receptor.
