# Supplementary material for: Residues 27T and 297A in VP2 contribute to the enhanced replication and pathogenicity of raccoon dog parvovirus
Source: J Virol. 2025 Sep 5;99(10):e01012-25. doi: 10.1128/jvi.01012-25 (PMC12548415; doi:10.1128/jvi.01012-25)
Supplement: Supplemental text — Report on genomic editing of TRVb cells via GenCRISPR-mediated engineering. [file jvi.01012-25-s0002.docx]

**Development of CHO-K1/Tfrc Knockout Cell Line**

**1. Summary**

This report summarizes the development of a CRISPR knockout cell line by using GenCRISPR™ gene editing technology. Based on the genomic sequences in database, target gene was analyzed and orientated via designing sitespecific guide RNA(s) (gRNA) for GenCRISPR™ system. By transient transfection of RNP (GenScript CRISPR singleguide RNAs: Cas9), the endogenous gene was targeted., resulting in consequential frameshift or removal of the designated fragment. Isogenic single cell clones were generated by cultivating the diluted transfected cells in 96-well plates and were identified by Sanger sequencing screening.

**2. Materials and Equipment**

**2.1 Materials**

| **Materials** | **Company** | **Cat.No.** |
| --- | --- | --- |
| DMSO | Sigma | D2650-100ML |
| Single guide RNA | GenScript | SC1969 |
| 0.25% Trypsin-EDTA | Gibco | 25200-072 |
| FBS | Gibco | A5669701 |
| Ham's F-12K (Kaighn's) Medium | Gibco | 21127022 |
| Opti-MEM | Gibco | 21127022 |
| Recombinant S. pyogenes Cas9 Protein | GenScript | SC1318 |

**2.2 Equipment**

| **Instrument** | **Company** | **Model** |
| --- | --- | --- |
| Electroporation device | Celetrix | D2650-100ML |
| Centrifuge | Eppendorf | SC1969 |
| Forma ClassII A2 Biological Safety Cabinet | Thermo | 25200-072 |
| Forma SeriesII Water Jacketed CO2 Incubator | Thermo | A5669701 |
| Microscope | Shanghai optical instrument import and export co., LTD | 21127022 |
| PCR thermal cycler | Hangzhou Bio-Gener Technology co., LTD | 21127022 |

**3. Information of the Deliverables**

| **Cell Name** | **Lot No.** | **Frozen date** | **Number (vial)** |
| --- | --- | --- | --- |
| CHO-K1 Negative cell* | C4498TZWG0-7/P2KC028 | 03/26/2025 | 2 |
| CHO-K1/Tfrc INDEL: +1/+1 Clone T1-49 | C4498TZWG0-7/P2KC028 | 04/21/2025 | 2 |
| CHO-K1/Tfrc INDEL: +1/-2 Clone T1-64 | C4498TZWG0-7/P2KC028 | 04/21/2025 | 2 |

* Negative cells are host cells transfected with control RNP complex consisting non-targeting sgRNA and Cas9

**Delivery quantity:** ≥ 1 × 10^6 cells/vial, 1 mL

**Storage condition:** -196°C

**Shipping condition:** -80°C Dry Ice

**Cell growth properties:** Adherent

**Complete growth medium:** Ham's F-12K (Kaighn's) Medium, 10% FBS

**Cryopreservation medium:** 90% FBS, 10% (V/V) DMSO

**4. Mycoplasma test**

The mycoplasma test was performed with MycoAlert™ PLUS Mycoplasma Detection Kit of Lonza.

| **Clone ID** | **Result** |
| --- | --- |
| CHO-K1 Negative cell | Negative |
| Clone T1-49 | Negative |
| Clone T1-64 | Negative |

**5. Experiments and Results**

**5.1 gRNA design and validation**

The gRNAs were designed to target the gene-of-interest. The gRNA cleavage efficiency was tested in cells by transient transfection and the gRNA selected for generation of the knockout cell line is shown below.

| **gRNA ID** | **Sequence** | **Cleavage efficiency^①^** | **Cell line** | **Comment** |
| --- | --- | --- | --- | --- |
| T1 | CCAGTGTCCGAAAACATAGA | 85% | CHO-K1 | Selected |
| T2 | ACAAAAAGACTGTGTAAGAC | 33% | CHO-K1 | Not Selected |
| T3 | CAATATAAGCGAGAAGATTG | 71% | CHO-K1 | Not Selected |

^①^ Cleavage efficiency is revealed by sequencing trace analysis with CAT tool (CRISPR analysis tool).

**5.2 Isogenic clone generation and Sanger sequencing screening**

**5.2.1 Genotype description**

The gRNA was transfected in the host cells and the transfected cells were plated in 96-well plates by limit dilution to generate isogenic single clones. The clones were picked from wells and screened by DNA sequencing to identify isogenic single clones with desired genotype.

| **Clone ID** | **INDELs^②^** | **Genotype** | **Data** |
| --- | --- | --- | --- |
| Clone T1-49 | +1/+1 | Full allelic knockout | Figure 1 |
| Clone T1-64 | +1/-2 | Full allelic knockout |  |
| CHO-K1 Negative cell | 0/0 | Full allelic knockout |  |

^②^ INDELs are the bps of insertion (+) or deletion (-) on alleles. “/” is used to separate INDELs among different alleles.

INDELs are revealed by sequencing trace analysis with CAT tool (CRISPR analysis tool).


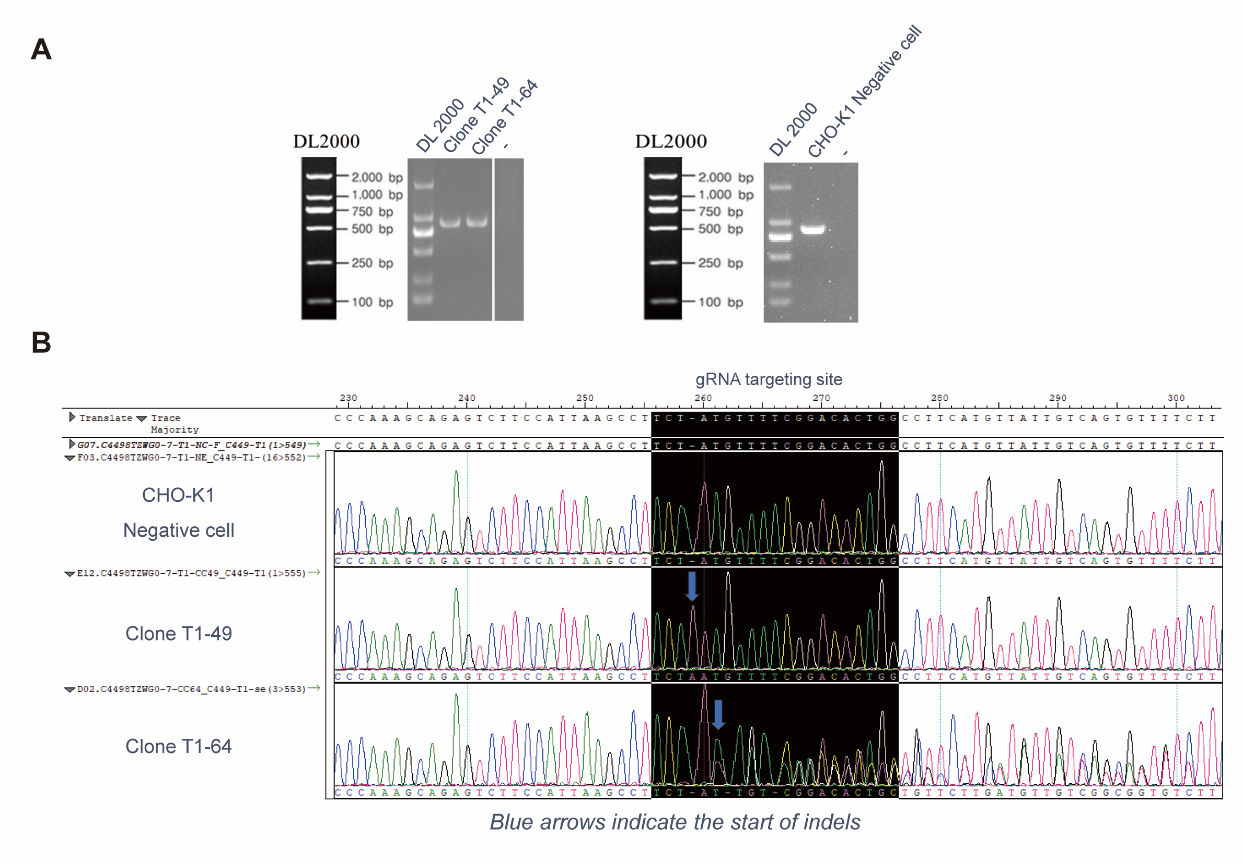


**Figure 1.** Genotyping of single cell clones

A. Map of electrophoresis of PCR products. B. Alignment of sequencing diagram.

Clone T1-49 and Clone T1-64 were confirmed as full-allelic knockout clones

**5.2.2 Primers for PCR**

Primers for Tfrc gRNA T1 site (5’>3’):

Forward: TCACTTGCCATTGACAGAATTGG

Reverse: CTCCGTTTCTGCCAGTCTTACA
